# Structure and Growth of the Leeward Kohala Field System: An Analysis with Directed Graphs

**DOI:** 10.1371/journal.pone.0102431

**Published:** 2014-07-24

**Authors:** Thomas S. Dye

**Affiliations:** Department of Anthropology, University of Hawai'i, Honolulu, Hawaii, United States of America; New York State Museum, United States of America

## Abstract

This study illustrates how the theory of directed graphs can be used to investigate the structure and growth of the leeward Kohala field system, a traditional Hawaiian archaeological site that presents an unparalleled opportunity to investigate relative chronology. The relative chronological relationships of agricultural walls and trails in two detailed study areas are represented as directed graphs and then investigated using graph theoretic concepts including *cycle*, *level*, and *connectedness*. The structural properties of the directed graphs reveal structure in the field system at several spatial scales. A process of deduction yields a history of construction in each detailed study area that is different than the history produced by an earlier investigation. These results indicate that it is now possible to study the structure and growth of the entire field system remnant using computer software implementations of graph theoretic concepts applied to observations of agricultural wall and trail intersections made on aerial imagery and/or during fieldwork. A relative chronology of field system development with a resolution of one generation is a possible result.

## Introduction

**Figure 1:**
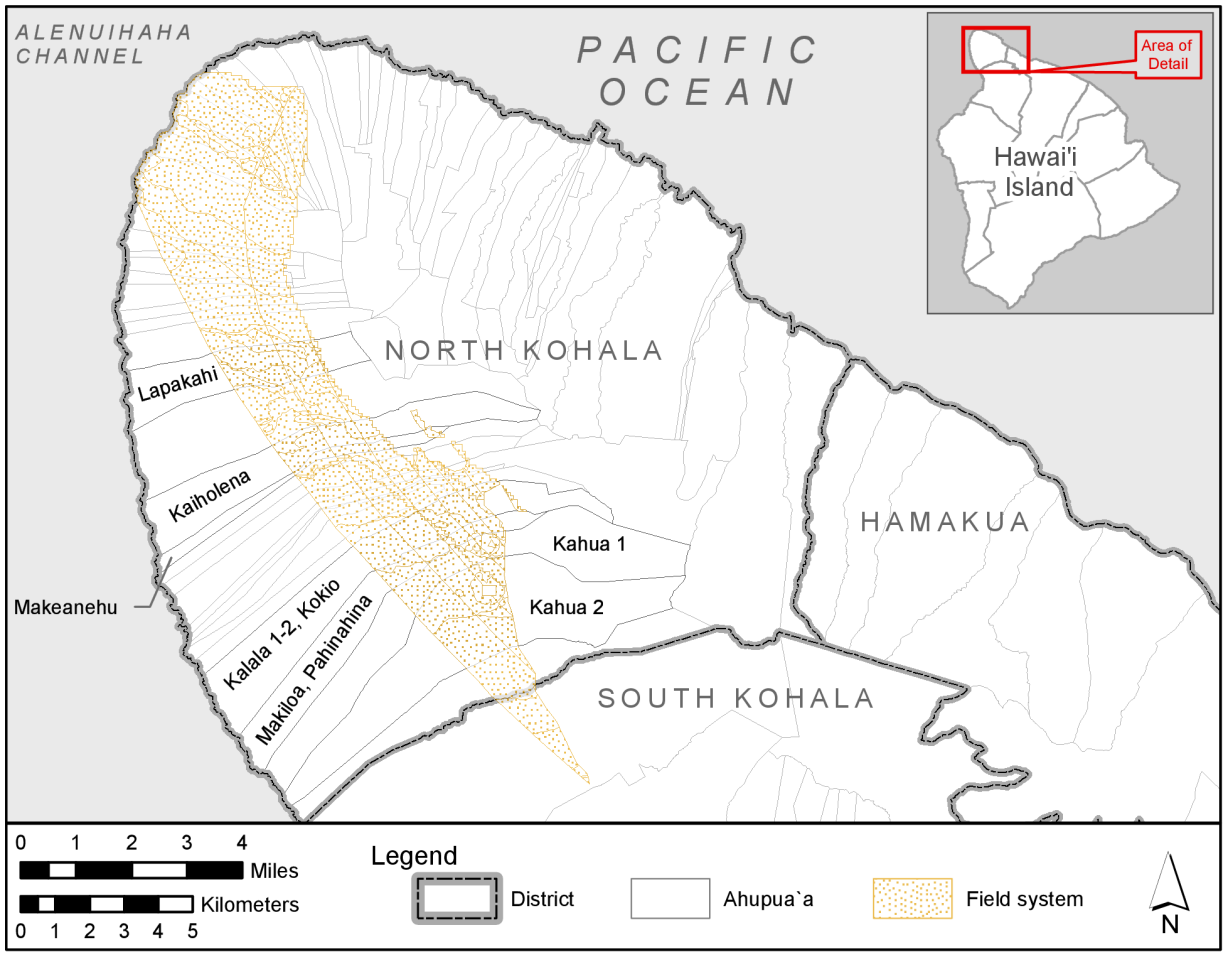
Location of the leeward Kohala field system. Also shown are the contact-era land divisions known in Hawai'i as *ahupua'a*. *Ahupua'a* land divisions where archaeological research in the leeward Kohala field system has concentrated are labeled.

**Figure 2:**
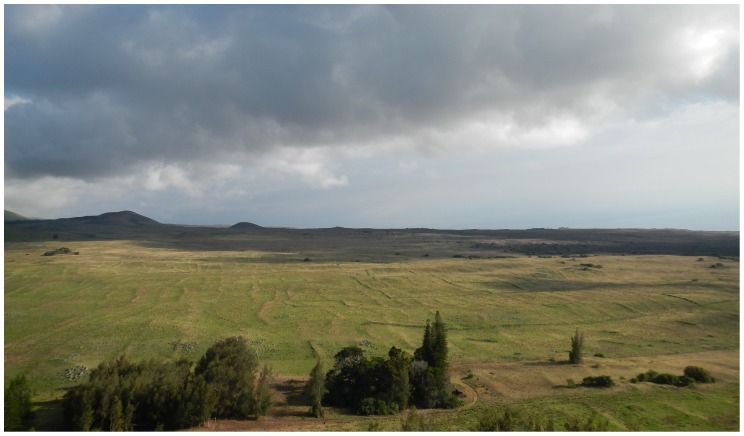
Photograph of a portion of the leeward Kohala field system. Note the agricultural walls running from the foreground to the background in this view looking south from Pu'u Kehena.

The leeward Kohala field system, a traditional Hawaiian rain-fed agricultural complex that covered 60 km^2^ on the leeward slopes of the Kohala Mountain on Hawai'i Island [1], offers the archaeologist an unparalleled opportunity to investigate relative chronology (Fig. 1). The traditional field system was converted to cattle pasture in the middle of the nineteenth century, but the agricultural walls and trails that once bound its gardens are visible today both on the ground and in aerial imagery (Fig. 2). In the decades since the field system was first identified as an archaeological entity [2], more than 570 km of agricultural walls—typically expressed as long, low earthen and rock mounds—have been identified [1]; the total length might be as high as 1,400 km [1]. The agricultural walls are cross-cut throughout the field system by more than 600 trails [3], which connected agricultural fields with coastal fishing villages and provided access to named subdivisions of the field system. This fabric-like structure, where trails provide warp for the weft of the agricultural walls, records relative chronological relationships at every intersection of an agricultural wall and a trail. Either a trail climbs over an older agricultural wall, or an agricultural wall ends at an older trail. By rough estimate, there are about 100,000 relative chronological relationships recorded by agricultural wall and trail intersections in the field system.

The structure of this set of chronological relationships has been investigated in three parts of the field system [3], which together comprise about two percent of its area, but the system itself has yet to be fully investigated. This paper proposes to use the theory of directed graphs [4] to investigate all of the relative chronological relationships recorded by the intersections of agricultural walls and trails in the leeward Kohala field system. It demonstrates that the growth and structure of two detailed study areas, one in the land of Lapakahi [5], [6] and the other in Kahua 1 and Pāhinahina [1], [7], can be investigated by modeling the chronological relationships of the agricultural walls and trails as directed graphs. These investigations reveal structure at various scales and yield a logically consistent relative chronology of field system development. The chronological results of both investigations differ from earlier proposals [1], [7]–[9], and the structural reasons for the differences are identified and described.

## The Leeward Kohala Field System

The primary crop grown in the field system was sweet potato. Direct evidence places the introduction of sweet potato to Hawai'i some three to five centuries after Polynesian settlement [10]. The plant underwent a remarkable radiation after its late introduction. By the early twentieth century, some 230 named varieties were known [11], the products primarily of rain-fed fields on the geologically younger islands [12] and colluvial sediments on the geologically older islands [13], [14]. The agronomic characteristics of the plant opened up vast areas of the geologically younger islands of Hawai'i and Maui for cultivation—one estimate is that the agricultural potential of Hawai'i Island more than tripled with the introduction of sweet potato [15]. A sophisticated program of ^14^C dating carried out at agricultural features within the leeward Kohala field system indicates the area was cultivated as early as the fifteenth century [7], probably in swidden gardens cleared from native forest. Later, the field system was developed with the agricultural walls visible on the surface today, which would have been planted with sugar cane when in use and likely increased yields by blocking the famously strong Kohala winds from damaging the sweet potato plants and reducing evapotranspiration [1]. Bayesian calibration of dating samples collected beneath agricultural walls indicates that efforts to increase production in the southern part of the field system began in the late seventeenth or early eighteenth century and that they continued in the eighteenth and early nineteenth centuries [16]. The period of intensification was relatively brief; the 67 percent highest posterior density region for one estimate is 100–189 years [16].

The sweet potatoes grown in the leeward Kohala field system supported development of pig herds reckoned by members of Captain Cook's crew as the largest they had encountered in the Pacific. In the late eighteenth century, the pig herds of the leeward Kohala field system were managed as wealth-assets by a line of *ali'i* based on leeward Hawai'i Island, whose rise to power was financed partially by this wealth [17]. When Cook was at Kealakekua Bay in 1779, the pig herds were controlled by the Hawai'i Island king, Kalani‘ōpu'u. Kalani‘ōpu'u was eventually succeeded by Kamehameha, who inherited the large Hawai'i Island pig herds. Kamehameha went on to unite the Hawaiian kingdom in the early historic period and the dynasty he established ruled the kingdom until King Kamehemeha V died in 1872. The legacy of the Kamehameha dynasty plays an important role today as Kamehameha Schools, the largest private landowner in the state with more than 363,000 ac. Kamehameha Schools provides educational services to more than 40,000 native Hawaiians annually and manages an endowment with a fair market value of $9.06 billion in 2011 [18]. This important piece of modern Hawai'i was built, in part, with the pig herds raised in the leeward Kohala field system.

The history of archaeological and ancillary investigations of the leeward Kohala field system was summarized recently [1] and the place of field system studies in the broader field of Hawaiian archaeology has been discussed in several recent books [19]–[21]. Research on the field system is continuing. Some recent studies include investigations of residential sites [22]–[24], religious structures [25]–[27], construction dates of agricultural walls and trails [16], ecological models of agricultural intensification [28], and agricultural infrastructure density using LiDAR imagery [29].

## Directed Graphs and Chronological Structure

The mathematical theory of directed graphs was developed to aid the investigation of the abstract notion of “structure.” It is “concerned with patterns of relationships among pairs of abstract elements” [4]. The theory itself makes no reference to the empirical world, but instead serves “as a mathematical model of the structural properties of any empirical system consisting of relationships among pairs of elements” [4]. The suitability of directed graphs for modeling chronological structure is widely recognized in archaeology through their correspondence with the Harris Matrix [30], [31].

This section describes how directed graphs are used to model the relative chronological relationships recorded by the intersections of agricultural walls and trails in the leeward Kohala field system, an effort that is conceptually similar to the extension of the Harris Matrix [32] to the recording of standing structures [33], and one that yields a total site matrix [34] that potentially captures all available relative chronological information. Following this, practical applications of three theoretical properties of directed graphs to the problem of relative chronological structure are illustrated. These include detecting logically impossible combinations of relationships using *cycles*, establishing relative ages of agricultural walls and trails by assigning *levels*, and identifying temporal discontinuities using the directed graph theoretic property of connectedness.

### Modeling Chronological Relationships in the Field System as a Directed Graph

The theory of directed graphs provides a precise vocabulary with which to describe the relative chronological structure of the field system. The chronological structure of the leeward Kohala field system may be thought of as chronological relationships on a set of agricultural walls and trails visible within the field system today.

A directed graph consists of one or more of a finite set of nodes and zero or more of a finite set of arcs. Each arc is specified such that it has a start node and an end node, which together indicate its direction. The agricultural walls and trails of the field system are represented as nodes and the relative chronological relation between an agricultural wall and a trail is represented by an arc. Although arc direction could be taken to mean either “younger than” or “older than”, the convention adopted here is that the start node of an arc is the older of the two and the end node the younger.

**Figure 3:**
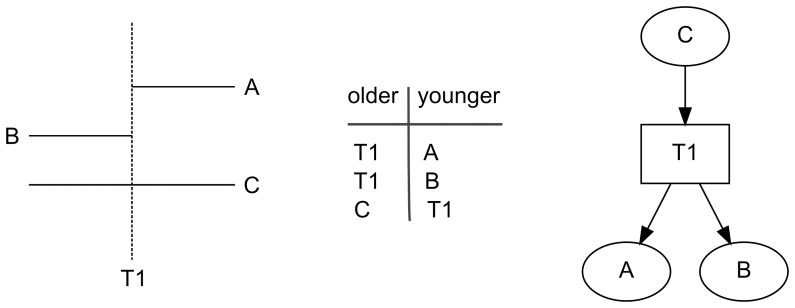
Relative chronological relationships of agricultural walls and trails. *Left*, map showing trail *T1* and agricultural walls *A*, *B*, and *C*; *middle*, table of chronological relationships shown on the map; *right*, picture of a directed graph of the chronological relations where ovals represent agricultural walls, the rectangle represents a trail, and arcs point from an older feature to a younger one.

Figure 3 is designed to illustrate the two fundamental chronological relations observable in the field system. It demonstrates how a map or an aerial image can be transformed first into a directed graph and then to a useful picture of a directed graph. The schematic map in Figure 3 shows the two chronological relationships possible between an agricultural wall and a trail. In one, a trail *t1* runs over pre-existing agricultural wall, *c*, which can be traced on either side of the trail. The other chronological relation is illustrated by agricultural walls *a* and *b*, which both end at their intersections with trail, *t1*, indicating that the trail was extant when the agricultural walls were built. These two relationships, first noted by Rosendahl [5], correspond to the Law of Superposition used in the interpretation of archaeological stratigraphy [32], [35]. Note that these chronological relationships are structural and that they apply regardless of how the agricultural walls might have functioned when in use.

The map can be transformed into a directed graph by focusing on the relations between agricultural walls and trails, rather than on the agricultural walls and trails themselves. One way to do this is to construct a two-column table of relationships (Fig. 3). In this table, the first column contains entries that correspond to the start of an arc, and the second column contains entries for the end. Each row of the table thus represents a complete arc. In the case of the field system, each row of the table has one entry for an agricultural wall and one entry for a trail. In the theory of directed graphs, arcs are represented as sets containing the start node and end node. The first row of the table in Figure 3 thus describes the arc (*t1*, *a*), the second row describes the arc (*t1*, *b*), etc.

The relations recorded in the table are also represented as a picture in Figure 3. The following conventions are used for constructing pictures of field system directed graphs. Nodes are represented as simple shapes, with trails represented by rectangles and agricultural walls represented by ovals. Arcs are represented as lines connecting two nodes; the start of an arc is unmarked, while the end of the arc is marked by an arrowhead. In a picture created with these conventions each arrow represents a chronological relationship established by observation and points from an older feature to a younger one. In the case of the field system, this observation can be made by an archaeologist in the field or by an analyst working with aerial imagery or a map of agricultural walls and trails. The two types of observation aren't mutually exclusive and any particular field system model might contain relationships established by a mix of field work and image analysis.

In the theory of directed graphs, the statement “agricultural wall *C* is older than trail *T1*” is expressed as node *C* is *adjacent to* node *T1*. The converse statement, that “trail *T1* is younger than agricultural wall *C*” is expressed as node *T1* is *adjacent from* node *C*. The *indegree* of a node is the number of nodes adjacent from it, and the *outdegree* is the number of nodes adjacent to it. In Figure 3, the agricultural wall *C* has an indegree of 0 and an outdegree of 1, trail *T1* has an indegree of 1 and an outdegree of 2, and agricultural walls *A* and *B* each have an indegree of 1 and an outdegree of 0. Because agricultural wall *C* has an indegree of 0 and a positive outdegree, it is called a *transmitter*. Agricultural walls *A* and *B*, which both have an outdegree of 0 and a positive indegree, are *receivers*. Trail *T1* is an *ordinary* node.

Because chronological relations are transitive, it is easy to see in Figure 3 that, because agricultural wall *C* is adjacent to trail *T1* and trail *T1* is adjacent to agricultural walls *A* and *B*, agricultural wall *C* is older than agricultural walls *A* and *B*. In the theory of directed graphs, the relationship between node *C* and node *A*, for example, is described generally as a *directed walk* and specifically as a *directed path*. A directed walk is a list of nodes and edges in order from the starting node to the ending node; a directed path is a directed walk in which no node appears more than once on the list. The *length* of a walk or a path is the number of arcs in it.

In the figure, node *A* is said to be *reachable* from node *C* because there is a directed path from node *A* to node *C*. The relative age of an agricultural wall and a trail is thus specified if the node for one of them is reachable from the other. In Figure 3 there is one pair of nodes whose relative age is undetermined; nodes *A* and *B* are not reachable from one another, so it is not possible to determine the relative ages of agricultural walls *A* and *B* with the evidence at hand.

### Detecting Errors: Cycles

**Figure 4:**
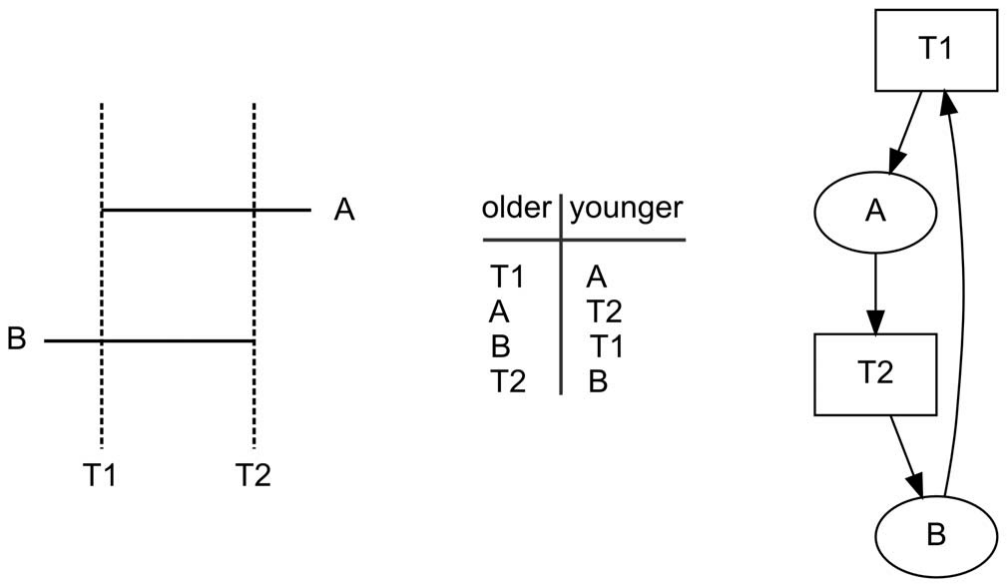
Example of a cycle. *Left*, map showing trails *T1* and *T2* with agricultural walls *A* and *B*; *middle*, table of the chronological relationships shown on the map; *right*, picture of a directed graph of the chronological relations, where ovals represent agricultural walls, rectangles represent trails, and arcs point from an older feature to a younger one.

Not all directed graphs represent valid chronological structures. In particular, a directed graph that contains a cycle cannot represent a system of chronological relationships [30]. In the theory of directed graphs, a *directed cycle* is a non-trivial directed walk that has the same first and last nodes. This situation is illustrated in Figure 4. The table of chronological relations records that agricultural wall *A* is younger than trail *T1* and older than trail *T2*, and that agricultural wall *B* is older than trail *T1* and younger than trail *T2*. In the picture of the directed graph (Fig. 4) it is easy to see that the graph lacks both transmitters and receivers; in fact in this simple directed graph every node has an indegree of 1 and an outdegree of 1. It is easy to show that, in this example, every agricultural wall and trail is older than itself. For example, trail *T1* is adjacent to, and thus older than, agricultural wall *A*; agricultural wall *A* is adjacent to, and thus older than trail *T2*; trail *T2* is adjacent to, and thus older than, agricultural wall *B*; and agricultural wall *B* is adjacent to, and thus older than, trail *T1*. Thus, trail *T1* is older than itself, a logical impossibility.

A routine check for cycles can be used to ensure that a directed graph is acyclic and thus logically represents a valid chronological structure.

In practice, cycles are introduced to a directed graph when there is an incorrect observation about the relative ages of a trail and an agricultural wall. Incorrect observations of this type are relatively easy to make with aerial imagery and also with maps of the field system, which so far have chosen to represent the agricultural walls and trails themselves without symbolizing the chronological relationships recorded by their intersections. Errors can arise in several ways. By chance, two late agricultural walls might abut a trail directly opposite one another; on a map or aerial imagery it might appear that a single agricultural wall ran under, and was thus older than, the trail. Or, the portion of an old agricultural wall on one side of a trail might be somehow obscured to aerial imagery, either through growth of vegetation, in which case it might show on a map, or by destruction during some later activity in the field system, in which case it might not appear on the map. In this case, only a direct observation of the intersection of the remaining agricultural wall and the trail might determine if there is evidence of a chronological relationship.

### Relative Ages of Agricultural Walls and Trails: Levels

The simple situations considered thus far conceal one of the benefits of using directed graphs to study the relative chronology of field system agricultural walls and trails. The chronological relationships in these simple situations might be read as easily from the map as from the table or the picture of the directed graph. In practice, when dealing with several trails and dozens of agricultural walls, the situation is often much more complex. It is in these more complex situations that the theory of directed graphs has great potential to benefit an investigation. Computer implementations of directed graph algorithms “scale up” well so that it is feasible to investigate very large structures, equal to or greater in size than the leeward Kohala field system.

**Figure 5:**
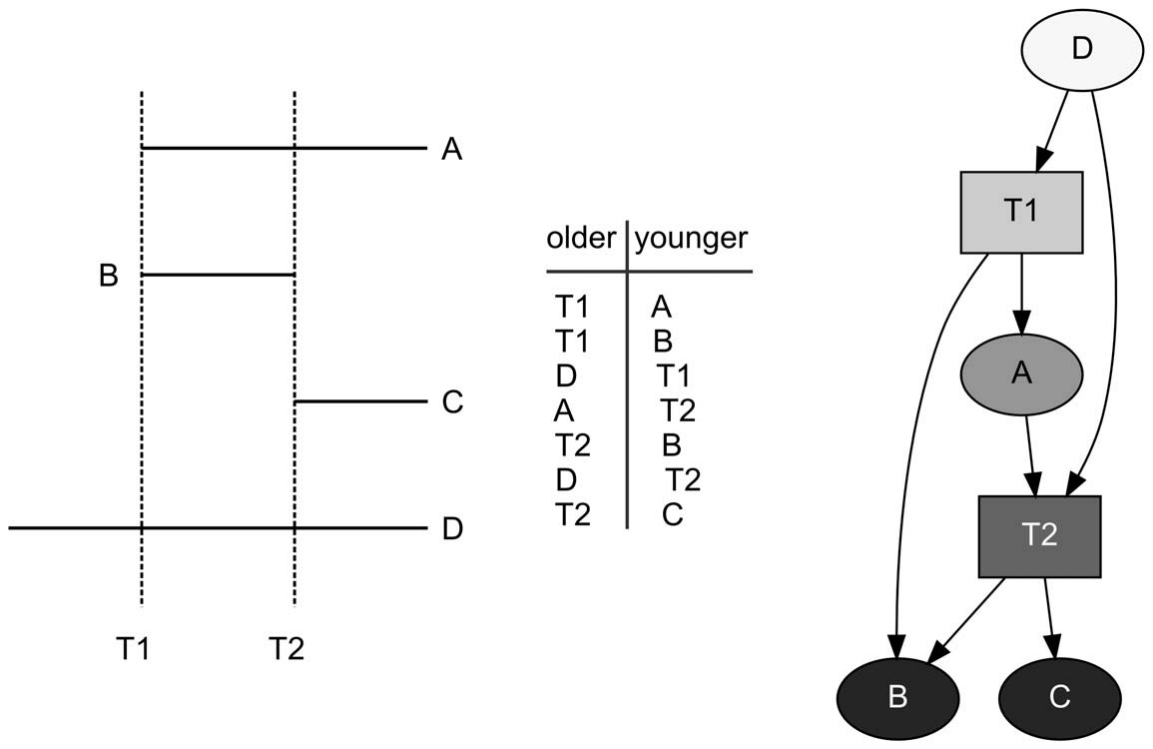
Level assignments. *Left*, map showing trails *T1* and *T2* with agricultural walls *A*, *B*, *C*, and *D*; *middle*, table of the chronological relationships shown in the map; *right*, picture of a directed graph of the chronological relations, where ovals represent agricultural walls, rectangles represent trails, and arcs point from an older feature to a younger one. Symbols in the directed graph are shaded by relative age; older features are lighter-colored than younger ones.

The simple situations considered so far have both included a single trail. With the addition of a second trail, the situation becomes more complex and difficult to comprehend (Fig. 5).

The theory of directed graphs makes it possible to express certain characteristics quantitatively. One example that is particularly useful for an investigation of chronological structure in general [36], and of the leeward Kohala field system in particular, is the concept of *level*. A graph has a level assignment when an integer is assigned to each node such that the integer assigned to each node is less than the integer assigned to any node adjacent to it. In this scheme, lower numbers are assigned to older features and higher numbers to younger features. In practice, each transmitter node is assigned the integer 0 and nodes adjacent to it are assigned the integer 1, the nodes adjacent to the level 1 nodes are assigned the integer 2, etc. In the field system, because each intersection that yields relative chronological information is of a trail with an agricultural wall, all the features of one type are assigned even levels, and all the features of the other are assigned odd levels.

On Figure 5, it is possible to place all the nodes from each level at the same vertical position on the graphic, so the node shading, also based on level, is redundant. Real-world graphs are sufficiently complex that this correspondence typically breaks down, and in these common situations symbolization based on level often proves useful.

The number of levels assigned in a graph of chronological relations in the field system is one greater than the length of the longest path in the graph [37]. Due to the fabric-like structure of relative chronological relations in the field system, the length of the longest path depends on the number of trails represented in the graph. Thus, in Figure 3, where there is only one trail, the length of the longest path is two and three levels can be assigned. In Figure 5, where there are two trails and trail *T2* is reachable from trail *T1*, the length of the longest path is four and five levels can be assigned.

### The Scope of Chronological Relationships: Connectedness

The number of levels that can be assigned in a graph of the leeward Kohala field system is an open structural question. If each of the more than 600 trails identified in the field system were reachable from a trail at one end of the field system, then it would be possible to assign more than 1,200 levels. This unlikely result would yield an extremely fine relative chronology. Given the rapidity with which the field system was developed, the average duration of a level in this instance would be measured in months rather than years.

In practice, such a situation is unlikely to be found. More likely, the field system contains chronological discontinuities such that nearby trails are reachable from a given trail but distant trails are not reachable. Chronological discontinuities such as these can be identified and investigated using the graph theoretic concepts of *subgraph*, *semi-walk*, and *connectedness* [30]. The definition of subgraph is straightforward. A graph, *S*, is a subgraph of another graph, *G*, if all the nodes and edges of *S* are also in *G*. A semi-walk in a directed graph is a walk in which the directions of edges are ignored; it ignores the chronological information encoded in the directions of the edges. There are three states of connectedness that a directed graph of chronological relations in the field system might exhibit, the first two of which potentially introduce chronological discontinuities. First, the directed graph might be *disconnected*, where one or more subgraphs do not share an edge with another subgraph. Second, the graph might be *weakly connected*, where there is a semi-walk that takes in all the nodes. There are no chronological discontinuities when a graph is *unilaterally connected*, such that for every pair of nodes, one is reachable from the other. Because graphs of chronological relations, such as the field system, are acyclic, it is not possible for them to be *strongly connected*. In a strongly connected graph any two nodes are reachable from each other—cycles are pervasive in a strongly connected directed graph.

**Figure 6:**
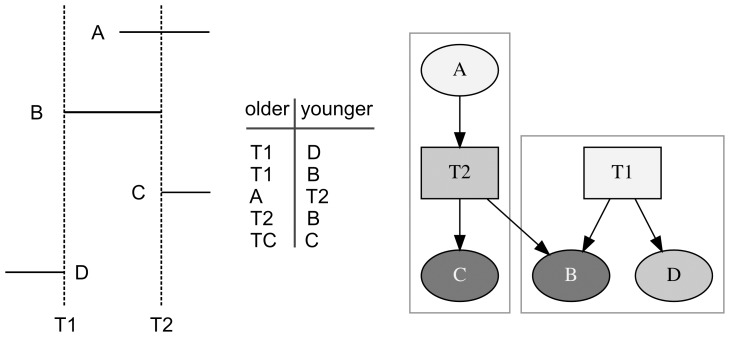
Unilaterally connected subgraphs of a weakly connected directed graph. *Left*, map showing trails *T1* and *T2* with agricultural walls *A*, *B*, *C*, and *D*; *middle*, table of the chronological relations shown in the map; *right*, picture of a directed graph of the chronological relations, where ovals represent agricultural walls, rectangles represent trails, and arcs point from an older feature to a younger one. The gray rectangles enclose unilaterally connected subgraphs. Note that agricultural wall *B* belongs to both subgraphs, but the picture shows it in only one.

Figure 6 illustrates two trails, *T1* and *T2*, and four agricultural walls, *A*, *B*, *C*, and *D*. Note that trails *T1* and *T2* are connected by a single agricultural wall, *B*. Because agricultural wall *B* is younger than both of the trails it is not possible to determine the relative ages of the trails. The graph of chronological relations is weakly connected, but contains two unilaterally connected subgraphs. This shows in the picture of the directed graph where a gray rectangle encloses the nodes and edges of each unilaterally connected subgraph. In the graph, trail *B* belongs to both of the unilaterally connected subgraphs; unilaterally connected subgraphs don't yield well-defined partitions of the parent graph. In the picture of the directed graph, trail *B* has been arbitrarily placed in the subgraph on the right-hand side of the picture and its inclusion in the subgraph on the left-hand side of the picture is indicated by the edge from trail *T2* that crosses the subgraph boundaries. The space between the gray rectangles is the temporal discontinuity. It is not possible to determine the relative ages of two nodes when they don't belong to the same subgraph.

## The Detailed Study Area at Lapakahi

The first detailed map of a portion of the leeward Kohala field system was drawn by Paul Rosendahl in 1970 using a plane table and alidade. The detailed study area, located in the traditional land unit of Lapakahi, was about 2.3 km long and 0.3 km wide [6]. Its boundaries were described as follows:

on the north, an inland-seaward extending curbstone-lined foot trail (Trail III); on the south, a similar foot trail (Trail IV); on the east, the inland end, a fence line along the edge of the cattle pasture land; and on the west, the seaward end, the lower distribution limits of the agricultural field unit boundaries. [6]

The drafted map, folded in a pocket inside the back cover of the dissertation [5], measured 11 by 66 in. The map was later published in a journal article where it was split over eight pages, making it difficult to get a sense of the complete map [6]. A digital version of the complete map is included as Figure S1.

The map includes a wealth of information on the spatial arrangement of agricultural walls and trails. In addition to the agricultural field units and trails, major features symbolized on the map include four forms of residential structures, three types of garden area, a variety of animal enclosures and pens, water catchments, ceremonial structures, and burial mounds and platforms. Agricultural walls, labeled “field boundary” on the map, include “earthen embankments,” which are most common at the inland end of the detailed study area, and “embankments with low piled stones,” which are dominant at the seaward end.

The two trails that define the northern and southern boundaries of the detailed study area are forks of a single trail with a terminus at the coastal village of Koai'e. Heading inland, both of the trails fork again about 0.5 km into the detailed study area. The two forks of the northern trail parallel one another at a distance of about 75 m and both exit the detailed study area at its eastern end. The two forks of the southern trail, however, merge repeatedly, creating a complex pattern. The final merge heading inland takes place just before the trail crosses a gully, and the trail exits the detailed study area as a single trail. Thus, at its inland end the detailed study area is two trails wide and at its seaward end it is three trails wide. Several shorter trail segments were mapped wholly within the detailed study area, so that middle sections of the detailed study area include as many as six trails.

On the basis of a settlement pattern analysis, Rosendahl hypothesized that the detailed study area represented two local community groups, one that occupied the area between the two branches of the northern trail, and another that occupied the area south of this to the boundary of the detailed study area. This analysis was influenced by the locations of two religious structures near the inland end of the detailed study area, one adjacent to the northern boundary trail and the other adjacent to the southern boundary trail. Based on observations of these two structures and a third structure located south of the detailed study area, Rosendahl identified four locational characteristics of the religious structures:

- 4.9–5.0 km from the coast;
- 1,175–1,200 ft. above sea level, with sweeping views;
- one per land unit; and
- approached by a recognized trail.

The map includes several walled enclosures that can be confidently assigned to the historic era on formal grounds. Rosendahl noted that

the enclosed garden areas represented an attempt to cultivate a restricted and protected portion of land under conditions of stress … caused by the large numbers of domestic and feral introduced animals, such as cattle and sheep, which were permitted to forage at will throughout the Lapakahi area. [5]

An historic-era use is also indicated by the presence at enclosed residential structures of artifacts made of metal, ceramic, and glass. There are three places in the detailed study area where these historic-era enclosures are found: at the inland end near the pasture land; along a gully near the middle of the detailed study area; and at the seaward end as part of a large complex of enclosures that extends south beyond the detailed study area. The walls of some of these historic-era enclosures appear to have been constructed on the earlier agricultural embankments, but wherever they occur the inference of relative chronological relationships of the earlier field system features is complicated and made difficult.

Although Rosendahl noted the relative chronological relationships recorded by the intersection of agricultural walls and trails in a general way, they are not symbolized on the map. The decision to bound the map on the north and south by major trails greatly reduced the utility of the map for investigating relative chronology. The map does not indicate which of the agricultural walls run under and are older than these trails and which of the agricultural walls abut and are younger than the trails. Thus, at the seaward end of the detailed study area, where only two trails are present, it is not possible to work out any relative chronological relationships from the plane-table map. At the inland end, where there are three trails, only the middle trail can be related chronologically to the agricultural walls. In order to make full use of the plane-table map for relative chronology, it is necessary to supplement it with observations of trail and agricultural wall intersections, either in the field or on aerial imagery. The first widely available aerial imagery useful for this purpose was added to Google Earth in January 2013, and this imagery is the basis of the supplemental observations used in this paper.

A dozen years after Rosendahl completed his dissertation, the potential for research into the relative chronology of field system features was illustrated with a schematic map showing three phases in the development of an approximately 1 km portion of the agricultural field system at Lapakahi [8], [9]. In the first phase, the trails at the north and south boundaries of the map were established along with 15 agricultural walls, most of which ran the full distance between the two trails. During the second phase, the fork of the northern trail was established along with about 40 relatively long agricultural walls. In the final phase, the various branches of the trail at the southern boundary were established along with about 40 short agricultural walls. This schematic map served as a model and provided the data for a later effort that set out five ordering rules whose application replicates the three-phase developmental sequence “almost exactly” [3].

**Figure 7:**
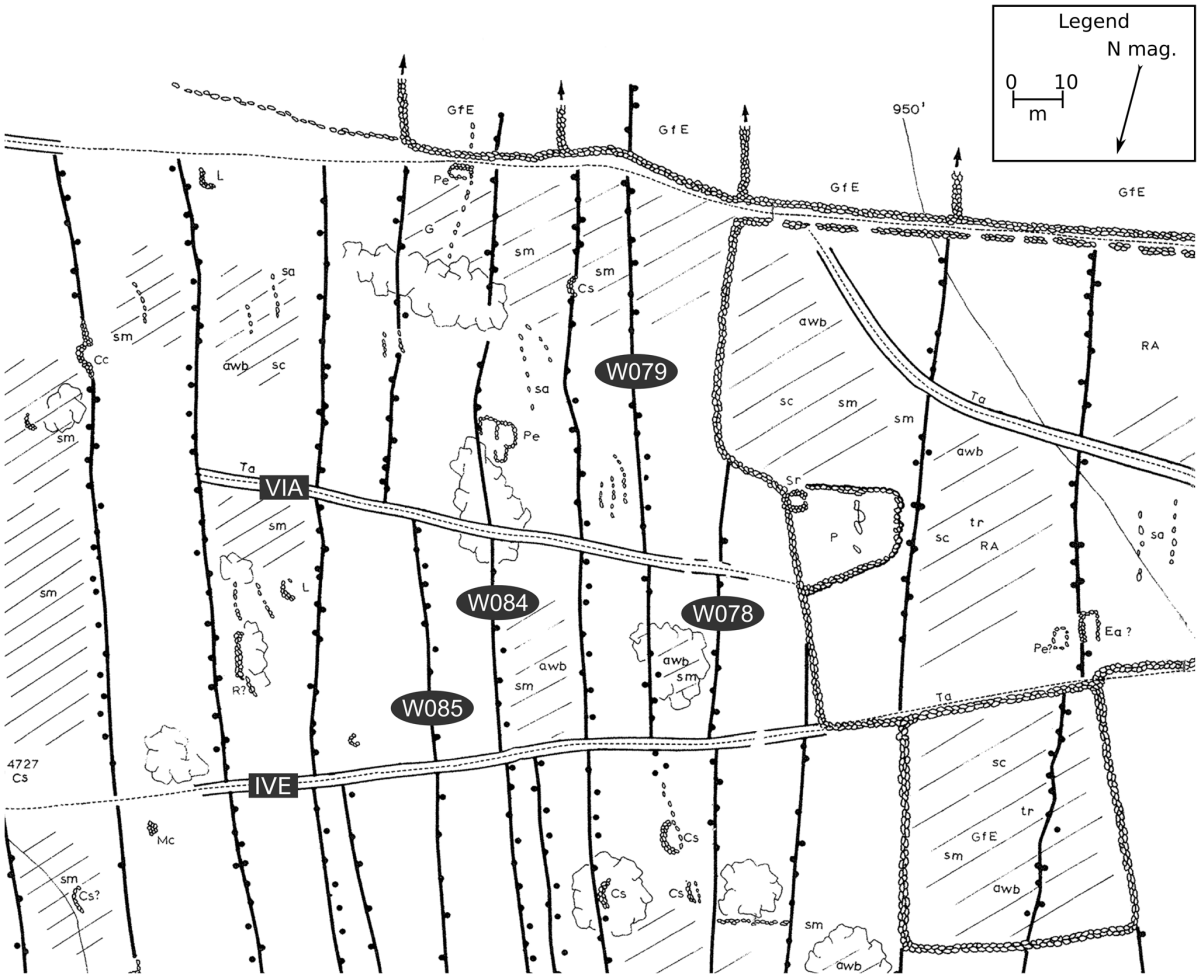
Cycles in a portion of the map of the Lapakahi detailed study area. See the text for an explanation of the map. The full map of the Lapakahi detailed study area is included as Figure S1, which includes a legend.

The chronological structure of the agricultural walls and trails of the Lapakahi detailed study area was investigated with directed graphs. A first step involved assigning a unique identifier to each of the agricultural walls and trails. In the case of agricultural walls, this meant that determinations were made about whether two agricultural wall segments, one on either side of a trail, passed under the trail and thus belonged to the same agricultural wall, or abutted the trail and thus belonged to different agricultural walls. These determinations, made by observing maps, aerial imagery, and the agricultural walls and trails themselves were typically straightforward and it was possible to model large portions of the map without introducing cycles. Problems were encountered most frequently in areas with evidence for historic-era wall building. A good example is near the seaward end of the map near the southern boundary (Fig. 7). Here, there are two trails, IVE and VIA, that share relationships with six agricultural walls. Trail IVE is one of the many branches of the southern boundary trail. Trail VIA is a short, disconnected trail segment that is not connected to other trails nearby. It terminates at its western end near a large historic-era enclosure and at its eastern end at an agricultural wall. Agricultural walls W078, W079, and W084 appear on the map to abut trail IVE and run under trail VIA, indicating that trail VIA is younger than trail IVE. However, agricultural wall W085 appears to abut trail IVE and run under trail VIA, which introduces a cycle. In this instance observations of Google Earth imagery don't help. The solution was to divide agricultural wall W085 in two at the trail IVE intersection. This tactic eliminates the cycle and makes the model logically correct, but does it reflect the actual relative chronological relationships? This example clearly points out the potential utility of symbolizing the relationships between agricultural walls and trails on a map of this type. As it stands, it is not possible to be confident that this part of the model of relative chronological relationships is historically accurate. Additional observations are clearly indicated.

Assigning unique identifiers to the trails presented a different challenge. It is sometimes asserted that the relative ages of two trail segments can be established by their relation to a third trail segment.

Branching trails are younger than the trails from which they branch. Branching is identified by the division of a single linear alignment into two sections, a stem and a branch. Branches diverge from and therefore become visible based on the heading and orientation of the stem. [3]

**Figure 8:**
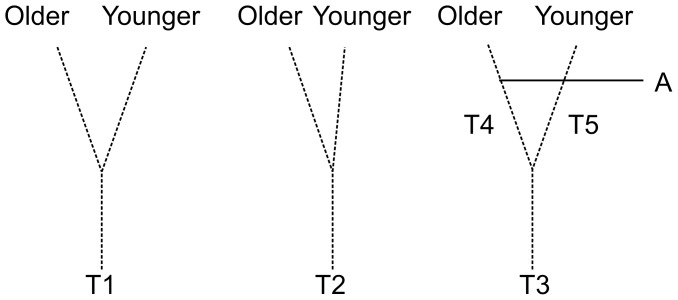
Relative ages of trail segments. *Left*, map showing a trail, *T1*, with two branches that diverge from the stem at similar angles; *middle*, map showing a trail, *T2*, with a bend and a younger branch oriented closely to a portion of the stem; *right*, map showing trail segments *T3*, *T4*, and *T5* and an agricultural wall, *A*, whose relationships with *T4* and *T5* establish that *T4* is older than *T5* (see fig. 3).

One problem with this formulation is that it assumes orientation offers an unambiguous way to distinguish a branch from a stem. However, it is not possible to distinguish relative ages when two trail segments diverge equally from the orientation of the stem (Fig. 8 *left*). Also, orientation potentially yields an incorrect result when a younger segment branches from a bend in a trail (Fig. 8 *center*). Given these potential ambiguities, it makes sense to assign unique identifiers to trail segments between forks and determine the relative ages of trail segments by their shared relationships to agricultural walls (Fig. 8 *right*).

**Figure 9:**
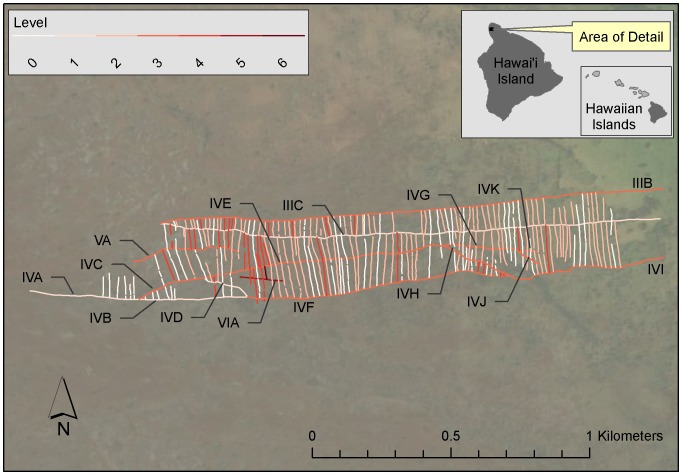
Level assignments in the Lapakahi detailed study area. The trails are labeled. See File S1 for a map of features that includes agricultural wall labels. See Table S9 in File S3 for the model of relative chronological relationships used to create the map. A picture of the directed graph of the relative chronological relationships in the Lapakahi detailed study area is included as Figure S2.

The level assignments identified four level 1 trail segments, ten level 3 trail segments, and one level 5 trail. The sequence of trail development shows most clearly at the inland end of the detailed study area (Fig. 9). Here, the middle of the three trails is the oldest, and the trails that mark the northern and southern boundaries of the detailed study area were established subsequently. Google Earth imagery in concert with the plane-table map yields quite a bit of evidence for the relative age relationships of these trails. More than three dozen agricultural walls that abut the middle trail on the plane-table map can be observed on Google Earth imagery to run under the trail at the northern boundary. Similarly, more than two dozen agricultural walls that abut the middle trail on the plane-table map can be observed on Google Earth imagery to run under segments of the trail at the southern boundary. The level assignments indicate that the various branches of the southern trail were established pene-contemporaneously at a late period in field system development. The assignment of the seaward end of the southern boundary trail to an early level appears to be due primarily to the rudimentary nature of the agricultural walls here, which hinders the observation of relative chronological relations. The early level assignment at the seaward end of the southern boundary trail is likely to be a matter of data quality, rather than an indication of historical process.

The two religious structures identified by Rosendahl in the Lapakahi detailed study area are located adjacent to the younger trails at the northern and southern boundaries of the detailed study area. Both of the religious structures are associated on their seaward, downhill sides with residential features characterized by high-walled rectangular enclosures. Rosendahl noted that these rectangular residential enclosures “frequently yielded surface finds of historic artifacts and bones of introduced mammals” [6] and hypothesized that the structures themselves belonged to the historic period. No religious structure associated with the older trail was identified, but a residential enclosure feature, similar in size to the residential enclosures associated with the religious structures, and located at a slightly higher elevation, was recorded adjacent to the middle trail. It has a small square enclosure inside the southeast corner at the uphill end of the larger enclosure. Interestingly, all three of these residential enclosures appear to pre-date construction of some agricultural walls in the vicinity. Each of the residential enclosures is abutted by one or more agricultural walls, and the plane-table map does not show any trace of the agricultural walls inside the enclosures. This pattern contrasts with various other large enclosures in the Lapakahi detailed study area which were built after the agricultural walls that can be clearly identified in the interiors of these enclosures.

The long, narrow shape of the detailed study area at Lapakahi contains too few trails to yield a high resolution relative chronology. The analysis with directed graphs identified seven levels of agricultural walls and trails in the detailed study area at Lapakahi, four of which are agricultural walls. The earliest features are 48 agricultural walls assigned to level 0. Many of these level 0 agricultural walls are short stubs at the seaward and inland ends of the detailed study area and their assignment to level 0 might be due to data quality issues, rather than historical process. The relatively few long level 0 agricultural walls, those that run under the early trail, IIIC, appear to be more common at the inland end of the map than they are where the trail branches. There were 110 agricultural walls assigned to level 2, 34 to level 4, and one to level 6. The level 6 agricultural wall, W237, was assigned late in the analysis in order to break a cycle associated with trail segment VIA; perhaps field observations will correct the model in the vicinity of the historic-era walls at the seaward end of the detailed study area.

## The Detailed Study Area at Kahua 1 and Pāhinahina

The map of the detailed study area at Kahua 1 and Pāhinahina is a 600 by 500 m portion of a larger detailed study area. It was designed to illustrate the locations of ^14^C dates used in an analysis of absolute chronology [7]. On this map, agricultural walls and trails are both indicated by lines symbolized to indicate assignment to one of five building phases. Other types of cultural feature and information on the natural environment are not shown on the map.

The mapped area is located immediately seaward of two cinder cones, Pu'u o Lani and Pu'u Lepo (see File S1). Just south of these two is the large cinder cone, Pu'u 'Aiea. These cinder cones are the source of several gullies, one of which originates at Pu'u o Lani and forms the northern boundary of the map, and another that originates inland of Pu'u 'Aiea and runs diagonally through the detailed study area, entering near the southeast corner of the map and exiting the seaward end near the middle of the map.

Google Earth imagery shows two parts of the mapped area where the agricultural walls have been disturbed by historic-era activities (see File S1). About two-thirds of the way to the inland end of the map is a rectangular area about 330 by 140 m that is dark green; this area appears to be the northern end of a rectangular strip more than 1.5 km long that runs north from Pōhakuloa Gulch over the Pu'u 'Aiea cinder cone and into the detailed study area. Farther seaward, in the southern half of the detailed study area, activities associated with cattle ranching have created a patch of bare dirt about 200 m^2^, within and around which agricultural walls are either missing or difficult to distinguish. The density of agricultural walls recorded in both of these disturbed areas is lower than surrounding areas.

The five building phases symbolized on the schematic map follow the Lapakahi example [8], [9] and assign both trails and agricultural walls to each of the phases except the first, which includes only agricultural walls. The building phase sequence was generated by application of a set of rules, the latest of several sets of rules that have been proposed for working out a relative chronology of agricultural walls and trails [3], [38], [39]. The rule sets have evolved over time as data have improved and as previous results were found to be unsatisfactory in some way [1]. The trail assigned to the earliest phase marks the boundary between the land of Pāhinahina on the north and the land of Kahua 1 on the south. “Once this trail was established, agricultural development was independent on either side of it” [1], and the building phase map shows a marked difference in the ages of agricultural walls and trails north and south of the boundary trail. The building phase map places agricultural walls in the land of Pāhinahina in Phases 1–3 and agricultural walls in the land of Kahua 1 mostly in Phases 3–5, indicating a generally early development of the field system in Pāhinahina followed later by development in Kahua 1 [1], [7].

The analysis of the Kahua 1 and Pāhinahina detailed study area with directed graphs was both easier and more difficult than it was at Lapakahi. One characteristic of the Kahua 1 and Pāhinahina detailed study area that made the task easier is that none of the trails branch within the area of the map. In addition, the building phase map typically indicates the temporal relationship of an agricultural wall to the trails it intersects, which greatly eases the task of assigning relative ages to features. Initially, the intersections of agricultural walls W060 and W063 with trails T004 and T006 introduced a cycle, but in correspondence Thegn Ladefoged indicated that field observation placed trail T006 older than agricultural wall W060. When this relationship was corrected, the cycle was eliminated. The main difficulty, which could not be solved with the materials at hand, was the relationship of the southern-most trail with the agricultural walls that intersect it. The problems appear to be concentrated at the seaward end of the map, where observations of Google Earth imagery indicated chronological relationships that introduced cycles. A consistent method for distinguishing correct from incorrect relationships could not be worked out. This appears to be an instance where field observations will be required to work out relative chronological relationships.

**Figure 10:**
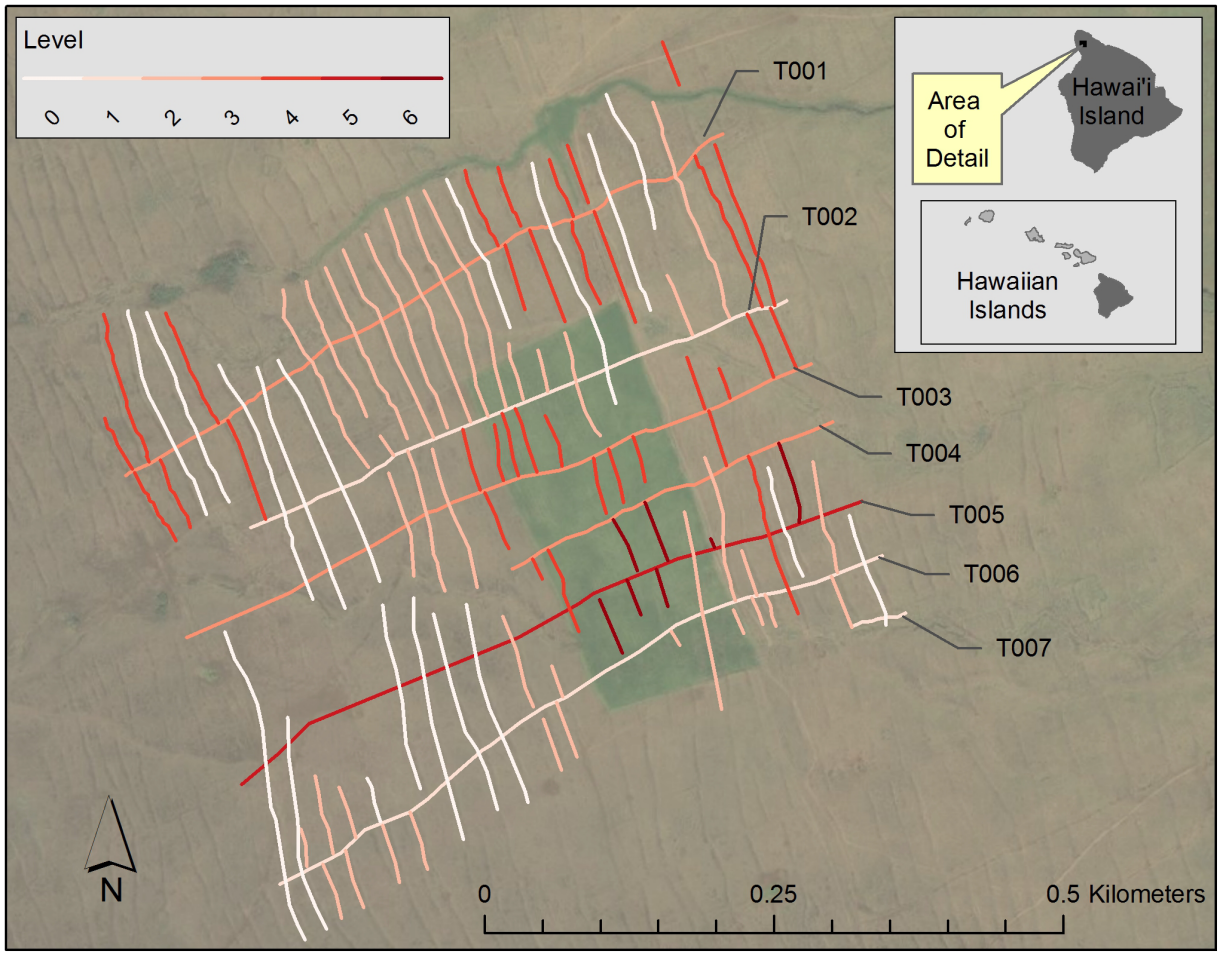
Level assignments in the Kahua 1 and Pāhinahina detailed study area. The trails are labeled. See File S2 for a map of features that includes agricultural wall labels. See Table S10 in File S3 for the model of relative chronological relationships used to create the map. A picture of the directed graph of the relative chronological relationships in the Kahua 1 and Pāhinahina detailed study area is included as Figure S3.

The analysis with directed graphs identifies three early trails as belonging to level 1 (Fig. 10). One of these is trail T002, which marks the Pāhinahina and Kahua 1 boundary. Another is trail T006 in Kahua 1, and lastly, the short trail segment, T007, also in Kahua 1. As already noted, there is contradictory evidence that might indicate trail T006 is younger than the trail immediately south of it. Also, the level assignment of trail T007 is based on a single intersection, which cautions against placing much interpretive weight on it. Three trails, one in Pāhinahina and two in Kahua 1, are assigned to level 3. Trail T005 in Kahua 1 is assigned to level 5 and appears to be the youngest trail in the map.

Of the eighteen agricultural walls assigned to level 0, several are short segments for which only one intersection was recorded; the level assignment of these short agricultural wall segments might not reflect history. The longer level 0 agricultural walls with more than one intersection are concentrated at the seaward end of the map in both Pāhinahina and Kahua 1. There is one long level 0 agricultural wall near the inland end of the map in Pāhinahina. Thirty-nine agricultural walls were assigned to level 2, most of them in a cluster immediately inland of the level 0 agricultural walls in Pāhinahina and Kahua 1, but with others scattered over the rest of the map. The 34 agricultural walls assigned to level 4 typically occur inland of the level 2 agricultural wall cluster, but are also found at the seaward end of Pāhinahina where they mix with level 0 agricultural walls. The seven level 6 agricultural walls are all associated with the late trail T005 in Kahua 1.

The map produced by the directed graph analysis indicates that the boundary trail doesn't divide the detailed study areas into an older northern part in the land of Pāhinahina and a younger part in the land of Kahua 1. The youngest agricultural walls and trails assigned to levels 5 and 6 are in Kahua 1, but the two halves of the map are otherwise similar in their level assignments. This is due, in part, to the fact that a graph of the map is weakly connected, comprising two unilaterally connected subgraphs. The boundary between the subgraphs is marked at its seaward end by the deep gully that cuts through the detailed study area. Near the middle of the map, the division exits the gully and runs between the two level 3 trails, T003 and T004. Thus, the northern subgraph takes in all the agricultural walls and trails in Pāhinahina along with trail T003 and its associated agricultural walls in Kahua 1. The southern connected component takes in all the agricultural walls and trails south of the gully along with a set of mostly recent features north of the gully at the inland end of the map.

Observations of Google Earth imagery indicate that the southern part of the detailed study area is the northern edge of a large, relatively unbroken, system in the lands of Kahua 1 and Kahua 2. The field system in Kahua 1 and Kahua 2 is wide and it appears to be serviced by several trails. These characteristics suggest that it might yield a high resolution relative chronology.

The overall impression from the map of levels is that agricultural walls and trails in the Kahua 1 and Pāhinahina detailed study area developed from the seaward end to the inland end, with areas near the gully developed later than flat lands away from the gully (Fig. 10).

## Discussion

The fabric-like structure of the leeward Kohala field system, which covers an area of 60 km^2^ and exposes approximately 100,000 stratigraphic relationships to investigation with aerial imagery and surface survey, presents an unparalleled opportunity to investigate relative site chronology. Paul Rosendahl's [5] insight that the intersections of agricultural walls and trails indicate superpositional relationships set the foundation for a structural analysis of field system development with directed graphs. Although a Harris Matrix was not produced during the directed graph analysis of the agricultural walls and trails reported here, the analysis is conceptually identical to the extension of the Harris Matrix to standing structures, and it yields the information needed to integrate the developmental chronology of surface features with the stratigraphic and dating information recovered during archaeological excavation to produce a total site matrix [34].

The relative chronological information yielded by the total site matrix for the Pāhinahina and Kahua 1 detailed study area was used in a Bayesian calibration of radiocarbon dates stratigraphically associated with agricultural walls and trails [16]. The Bayesian calibration benefited strongly from the information in the total site matrix, which was derived by hand rather than with the aid of computer software, and it yielded more detailed estimates of the tempo of field system development than an earlier ad hoc interpretation [7]. With the aid of computer software, the total site matrix approach might be extended to other parts of the field system as part of a project to document spatial variability in field system development. Currently, the hypothesis that development of the leeward Kohala field system was constrained by environmental characteristics is based on an ad hoc interpretation of the radiocarbon dates from the Pāhinahina and Kahua 1 detailed study area and a handful of dates on unidentified wood charcoal from the environmentally-favored northern end of the field system [7] whose potential for in-built age renders them unsuitable. This important hypothesis deserves testing with dates on suitable materials interpreted with a Bayesian calibration that uses an informative prior based on the total site matrix.

Investigation of the Lapakahi detailed study area map, augmented by observations of Google Earth aerial imagery, revealed a developmental sequence substantially different from the one indicated by ad hoc methods [3], [8], [9]. Here, the long, narrow shape of the detailed study area map together with the decision to bound the map by trails on the north and south magnified the impact of boundary problems that are common to spatial analyses. Once these boundary problems were mitigated by observing the intersections of agricultural walls with the two boundary trails on Google Earth imagery, abundant evidence could be mustered to show that the two trails that bound the map are younger than the major trail in the middle of the map, and not older, as the ad hoc analysis had indicated. The correct ordering of the trail construction sequence makes it possible to add a fifth characteristic to the four noted by Rosendahl for the locations of religious structures; they were constructed adjacent to, and were served by, a *late* trail. This result, which suggests that temple construction is associated with later rather than earlier stages of field system development supports the short, late construction sequence for family-sized temples in the leeward Kohala field system yielded by a Bayesian calibration [25] and does not support the long construction sequence yielded by an ad hoc analysis [26].

The situation at the Pāhinahina and Kahua 1 detailed study area is somewhat different. Here, the hypothesis that agricultural walls and trails in the northern part of the map are older than those in the southern part [7] is not supported by the published evidence, which includes a temporal discontinuity. Thus, the published evidence can't support the argument that the contact-era *ahupua'a* land divisions were in place when the field system developed, and that their boundaries, often marked by trails, exerted an influence on the tempo of development. Statements that single out the boundary trails as important structural features of the field system are based upon an unsupported and likely flawed relative chronology and are potentially misleading. For instance, the statement that “[l]ater walls … are increasingly confined within … [land] boundaries, suggesting that as more boundary trails were established, agricultural walls did not usually cross them” [1] is true, but trivially so, because it is true for all trails, whether or not they mark land boundaries. There is currently no evidence for the idea that boundary trails had more influence on field system development than other trails. The history of *ahupua'a* land divisions has been an important archaeological topic for some time [40], and archaeologists interested in this history will want to investigate several land boundaries to marshal evidence for the historical role of boundary trails in field system development. The question calls for an analysis with directed graphs and full publication of the supporting data.

The fabric-like structure of the field system and the historical depth of the relative chronological information it records are only partially recognized by certain ad hoc analytic methods. This shows clearly when the number of levels yielded by the analysis with directed graphs is compared with the structural levels yielded by a GIS analysis of LiDAR data for the field system [29]. The analysis with directed graphs identified seven levels within each detailed study area. At the Lapakahi detailed study area, the number of levels was constrained by the narrow shape of the mapped area; expanding the map either north or south to include more of Lapakahi will likely increase the number of levels that can be distinguished. Similarly, the temporal discontinuity at the Pāhinahina and Kahua 1 detailed study area limited the number of trails that were reachable from one another and constrained the number of levels that could be distinguished there. Expanding this map to include a larger portion of Kahua 1 would likely increase the number of levels that can be distinguished. In contrast, the GIS analysis of LiDAR data distinguishes three levels of structure, labeled “expansion”, “segmentation”, and “intensification”, which represents a substantial loss of potential historical information. GIS analysis of the field system might better be based on level assignments made with directed graphs, which take into account the full range of relative chronological information recorded by the intersections of agricultural walls and trails.

The use of directed graphs makes it possible to identify cycles, which represent errors typically introduced by one or more incorrect observations of relative chronological relationships [30]. Cycles were identified in maps of both detailed study areas. One instance at the Pāhinahina and Kahua 1 detailed study area was resolved when the investigator consulted field notes to determine the correct stratigraphic relationship of an agricultural wall and trail. Another instance in the Lapakahi detailed study area (Fig. 7) was resolved somewhat arbitrarily by dividing an agricultural wall in two. In the third instance, at the southwest corner of the Pāhinahina and Kahua 1 detailed study area, no simple solution could be found and the southernmost trail on the map had to be eliminated from the analysis. These situations are precisely analogous to the problems faced by stratigraphers at any complex site and which are the motivation for the practice of building stratigraphic sequences while excavations are in progress [41]. During an excavation, the stratigrapher must go back to the source of a cycle and observe the deposits and interfaces more closely before they are destroyed by subsequent excavation or buried when the excavation is filled. There is less urgency in the leeward Kohala field system, where identification of a cycle might be considered part of a research design for a program of field survey or other form of close observation. The goal in the field system is the same as on an excavation—a record of reliable stratigraphic observations that yield a valid and correct site developmental sequence.

This discussion has focused on the deficiencies of certain ad hoc stratigraphic practices and on the use of directed graphs to identify errors, but it is important to recognize that an analysis with directed graphs opens opportunities, as well. One reason to expand archaeological observations of the field system is to search for areas with sufficient structure to yield high resolution relative chronologies. It is conceivable that one or more areas will yield a relative chronology with a resolution that approximates a human generation. Agricultural wall building in the field system likely took place over a period less than two centuries [16], or about 10 generations. Ten levels can be distinguished in a directed graph with a longest path of length 9, which corresponds to an area where a young trail is reachable from an old trail through three trails of intermediate age. If an area like this can be found in the field system, then it will be possible to create a map that roughly tracks each generation's investment in the field system infrastructure.

A recent investigation of LiDAR data indicates that today, a century and a half since the field system was converted to cattle pasture, about a third of the field system has been lost to ranching and other activities and relative chronological relationships are difficult to determine in another third [28]. Now is the time to investigate the structure and growth of the leeward Kohala field system with directed graphs, before an unparalleled opportunity to investigate relative chronological relationships is lost to development.

## Supporting Information

Figure S1 **Paul Rosendahl's map of the Lapakahi detailed study area.**
(TIFF)

Figure S2 **Directed graph of the agricultural walls and trails in the Lapakahi detailed study area.**
(PDF)

Figure S3 **Directed graph of the agricultural walls and trails in the Kahua 1 and Pāhinahina detailed study area.**
(PDF)

File S1 **Level assignments in the Lapakahi detailed study area for plotting with Google Earth.**
(ZIP)

File S2 **Level assignments in the Kahua 1 and Pāhinahina detailed study area for plotting with Google Earth.**
(ZIP)

File S3 **A compendium** [44] **in Portable Document Format with detailed instructions how the reader can reproduce the analyses reported in this paper.** This file should be read with the Acrobat Reader application or a similar application capable of rendering the links in the third column of Table S2 in File S3.
(PDF)
