# Correction: Structure and Growth of the Leeward Kohala Field System: An Analysis with Directed Graphs

**DOI:** 10.1371/journal.pone.0109157

**Published:** 2014-09-22

**Authors:** The PLOS ONE Staff

File S1 is an accidental duplicate of File S2. The correct File S1 can be viewed below.

## Supporting Information

File S1 **Level assignments in the Lapakahi detailed study area for plotting with Google Earth.**
(ZIP)
