# Supplementary figures and images for: Structure and Growth of the Leeward Kohala Field System: An Analysis with Directed Graphs
Source: PLoS One. 2014 Jul 24;9(7):e102431. doi: 10.1371/journal.pone.0102431 (PMC4109926; doi:10.1371/journal.pone.0102431)

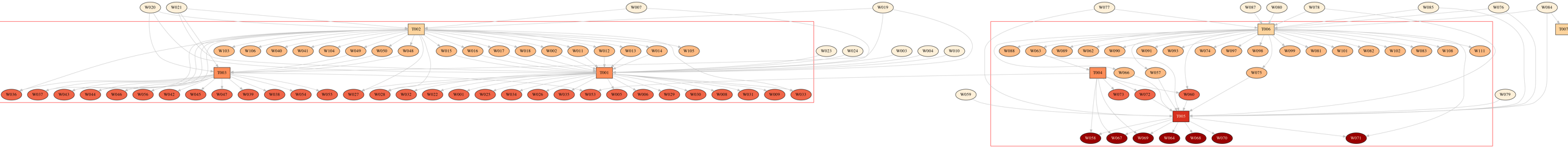

Supplement: Figure S3 — Directed graph of the agricultural walls and trails in the Kahua 1 and Pāhinahina detailed study area. (PDF) [file pone.0102431.s003.pdf]
