# Supplementary material for: Structure and Growth of the Leeward Kohala Field System: An Analysis with Directed Graphs
Source: PLoS One. 2014 Jul 24;9(7):e102431. doi: 10.1371/journal.pone.0102431 (PMC4109926; doi:10.1371/journal.pone.0102431)
Supplement: File S3 — A compendium [44] in Portable Document Format with detailed instructions how the reader can reproduce the analyses reported in this paper. This file should be read with the Acrobat Reader application or a similar application capable of rendering the links in the third column of Table S2 in File S3. (PDF) [file pone.0102431.s006.pdf]

Supporting Information for "Structure and Growth of the Leeward Kohala Field System: An Analysis with Directed Graphs"

Thomas S. Dye[1,*]

1 Department of Anthropology, University of Hawai'i, Honolulu, HI, USA

∗ **E-mail: tsd@tsdye.com**

List of Tables

S1 Open source software required to reproduce the analysis . . . 4
S2 Compendium contents . . . 5
S3 Tabular representation of a simple directed graph . . . 7
S4 Arguments passed to the source code block in Listing S2 . . . 7
S5 Tabular representation of a directed graph with a cycle . . . 10
S6 Arguments passed to the source code block in Listing S9 . . . 11
S7 Tabular representation of map with two trails . . . 12
S8 Tabular representation of connected components . . . 14
S9 Chronological relationships in the detailed study area at Lapakahi . . . 16
S10 Arguments passed to the source code block in Listing S13 . . . 26
S11 Chronological relationships in the detailed study area at Kahua 1 and Pāhinahina . . . 30

List of Source Code Block Listings

S1 Set up the Common Lisp environment . . . 6
S2 Noweb syntax for the Figure 3 source code block . . . 8
S3 Declare local variables . . . 8
S4 Add arcs to a graph . . . 8
S5 Conditionally plot the Figure 3 graph picture . . . 9
S6 Return a value from the source code block . . . 10
S7 Noweb syntax for the Figure 4 source code block . . . 10
S8 Conditionally plot the Figure 4 graph picture . . . 11
S9 Noweb syntax for the Figure 5 source code block . . . 12
S10 Conditionally plot the Figure 5 graph picture . . . 13
S11 Noweb syntax for the Figure 6 source code block . . . 14
S12 Conditionally plot the Figure 6 graph picture . . . 15
S13 Noweb syntax for the Lapakahi source code block . . . 26
S14 Conditionally plot the Lapakahi graph picture . . . 27
S15 Identify unilaterally connected subgraphs at Lapakahi . . . 28

S16 Plot the Lapakahi detailed study area graph picture 29
S17 Noweb syntax for the Kahua 1 and Pāhinahina source code block 34
S18 Conditionally plot the Kahua 1 and Pāhinahina graph picture 35
S19 Identify unilaterally connected subgraphs at Kahua 1 and Pāhinahina 35
S20 Plot the Kahua 1 and Pāhinahina detailed study area graph picture 36

Introduction

This document is a description of the data and analysis on which the conclusions of the paper, "Structure and Growth of the Leeward Kohala Field System: An Analysis with Directed Graphs," are based. It is a compendium [1] that contains i) 1. the text of the paper in Org mode format, 2. the bibliographic data in a BibTeX file, 3. the field system data used in the analysis, 4. the source code used to carry out the analysis and generate the illustrations, 5. graphics files for the figures in the article, 6. several large graphics that don't meet the journal's guidelines, and 7. Google Earth maps with which the reader can interactively explore the growth and structure of the leeward Kohala field system. With these materials, and a computer with certain open-source software packages properly installed (table S1), it is possible to reproduce the analysis [2] precisely and generate the Portable Document Format file that was submitted for publication. To make full use of the supplementary material, the reader's computer must also have software to display graphics files in `pdf`, `tiff`, and `jpeg` formats, as well as the freely available Google Earth application.

Much of the analysis is carried out with computer code written in the Common Lisp language. This source code has certain requirements to execute correctly. The source code currently runs on two open source implementations of Common Lisp, Steel Bank Common Lisp or Clozure Common Lisp, one of which must be installed on the user's computer. Most of the Common Lisp code contains calls to routines in the open source `graph.lisp` and `graph-dot.lisp` libraries. These libraries, along with the open source libraries on which they depend, must be installed on the reader's computer where the Common Lisp implementation can find them. Perhaps the easiest way to accomplish library installation is with the `Quicklisp` library manager, which will automatically install dependencies.

Directed graphs are drawn with the `dot` tool of the `Graphviz` software, which produces hierarchical or layered drawings of directed graphs. In practice, the `graph-dot.lisp` library produces `dot` source code,

Table S1. Open source software required to reproduce the analysis.

Software	Distribution	Installation
Emacs	GNU Emacs	See distribution instructions
	XEmacs	See distribution instructions
Lisp	Clozure Common Lisp	See distribution instructions
	Steel Bank Common Lisp	See distribution instructions
Lisp library manager	Quicklisp	See distribution instructions
Lisp interaction mode	Slime	See distribution instructions
Lisp graph library	graph.lisp	(ql:quickload "graph")
	graph-dot.lisp	(ql:quickload "graph-dot")
Dot	Graphviz	See distribution instructions
LaTeX	TeX Live (Linux)	See distribution instructions
	MacTeX (Mac OS X)	See distribution instructions
	proTeXt (Windows)	See distribution instructions
LaTeX packages	setspace	Required by *PLOS ONE*
	amsmath	Required by *PLOS ONE*
	amssymb	Required by *PLOS ONE*
	graphicx	Required by *PLOS ONE*
	cite	Required by *PLOS ONE*
	color	Required by *PLOS ONE*
	caption	Required by *PLOS ONE*
	minted	Used by the Supporting Information document
	longtable	Used by the Supporting Information document
	booktabs	Used by the Supporting Information document
	attachfile	Used by the Supporting Information document
	dtklogos	Used by the Supporting Information document
	paralist	Used by the Supporting Information document

which is written to a file that can be passed as input to `dot`. The `dot` software is capable of producing graphics output in about 25 formats; the two formats used in the analysis were Portable Document Format (pdf) for graphs used directly in the paper, and Scalable Vector Graphics (svg) for graphs used as components of larger graphics that were subsequently laid out with the open-source `Inkscape` software.

Field system maps were produced as figures for the paper and as Google Earth `.kmz` files for the supplementary material with the proprietary ArcInfo software. At this early stage in the analysis, ArcInfo software was used to produce illustrations of results achieved by the open-source software listed above but it was not used in the analyses themselves. It is anticipated that ArcInfo, or similar GIS software, will, in the future, productively investigate maps based on the results of directed graph theoretic analyses.

The paper was written and the analysis was carried out using the reproducible research facilities provided by the Org mode of Emacs, an open-source text editor [3,4]. Version 8 of Org mode is required. Org mode has been described as "a lightweight text markup language that enables intermingling of narrative text, data and analysis code in an active document" [5]. The Org mode file `lkfs-structure.org` and the BibTeX file `local.bib` are together sufficient to reproduce the analysis reported in "Structure and Growth of the Leeward Kohala Field System: An Analysis with Directed Graphs" and produce the

LaTeX source code file and the Portable Document Format file that were submitted to the publisher. Instructions for exporting the Org mode document to LaTeX and `pdf` are provided in the Org mode file.

The LaTeX file required by *PLOS ONE* must meet a particular specification. In order to meet this specification, the user's LaTeX distribution must include the seven packages indicated in Table S1; these packages are typically included in standard LaTeX distributions. Additional LaTeX packages are required to typeset this Supporting Information document, and these are listed in Table S1, as well.

The stand-alone files included with this compendium are listed in Table S2. The Org mode project file is a plain text file designed to be used with the Emacs text editor and an Org mode version 8.0 or later. The bibliographic database is a standard, plain text, BibTeX file used by the LaTeX document preparation system. Also included is the *PLOS ONE* bibliography style file. Graphic files for each of the ten figures in the article are also provided, so that a pdf copy of the paper can be generated by the user.

Table S2. Compendium contents.

Description	File name	Link to file
Org mode project file	`lkfs-structure.org`	
Project bibliographic database	`local.bib`	
PLOS ONE bibliography style	`plos2009.bst`	
Figure 1 graphic	`lkfs-map.pdf`	
Figure 2 graphic	`DSCN0147.jpg`	
Figure 3 graphic	`age-relations.pdf`	
Figure 4 graphic	`cycle.pdf`	
Figure 5 graphic	`trail-ages.pdf`	
Figure 6 graphic	`components.pdf`	
Figure 7 graphic	`929-cleaned-VIA.pdf`	
Figure 8 graphic	`trail-segments.pdf`	
Figure 9 graphic	`LKFS20131216.jpg`	
Figure 10 graphic	`LG-LKFS20131216.jpg`	

Set Up the Common Lisp Environment

The Common Lisp code runs in a particular environment. The source code block in Listing S1 sets up that environment. It must be run at the start of a Common Lisp session. In practice, the Emacs Lisp interaction mode is started with `M-x slime` and the code in Listing S1 is run by placing point within the

source code block and pressing C-c C-c.

Referring to Listing S1, Line 1 makes the graph-dot.lisp library available, which requires that the graph.lisp library is also available. Line 2 ensures that the code is executed in the namespace of the graph.lisp library and line 3 makes the symbols defined in the graph-dot.lisp library directly accessible.

```
1 (require 'graph-dot)
2 (in-package :graph)
3 (use-package :graph-dot)
```

Listing S1. Set up the Common Lisp environment.

Figure Source Code

This section describes the data and source code used to make the graph pictures shown in Figures 3, 4, 5, and 6. In each case, the full figure was constructed manually using the open-source scalar vector graphics editor Inkscape. The left and middle panels were drawn in Inkscape, and the right panel was imported as an svg file produced by the dot software from the code generated by the source code blocks described here.

The source code is described with the aid of the noweb syntax implemented by the Babel facility of Org mode. Noweb is a language-agnostic implementation of the literate programming tool web designed by Donald Knuth [6]. The idea behind literate programming is to prepare computer programs for human readers. Here, the use of noweb syntax structures the presentation and eases the descriptive burden somewhat, because several code chunks can be re-used once they have been first used and described.

Source Code for the Figure 3 Graph Picture

The figure is designed to show a schematic map, graph table, and graph picture side by side to illustrate the process of moving from observation, to model, and then to picture.

Data Table for Figure 3

The simple computer code for plotting the Figure 3 graph picture expects an input table in which each row represents a single arc. It further expects that the start of the arc is in the first column and the end of the arc is in the second column. Additional columns, if present, are ignored and the column heads are also ignored.

Table S3. Tabular representation of a simple directed graph.

Older	Younger
T1	A
T1	B
C	T1

Header Arguments for Figure 3

The Common Lisp source code block that plots the age relation data takes two header arguments that name and set local variables and one header argument that specifies the directory where the code will be executed (Table S4). The variable `arcs` is passed a reference to Table S3, and the other two header arguments are set according to the user's situation.

Table S4. Arguments passed to the source code block in Listing S2.

Argument	Value
:var arcs	an Org mode table of nodes where the first two columns are "Older" and "Younger"
:var out-file	a string holding the name of the output file name with suffix, e.g. "file.dot"
:dir	a path where the code will run and write its output

Structure of the Figure 3 Source Code Block

Listing S2 includes four noweb-style code chunks nested within a Common Lisp `(let)` special operator. The first step, at line 2, declares local variables. Then, at line 3, the arcs in Table S3 are added to the graph. If this step is accomplished successfully, then `dot` source code for the graph should be written to file (line 4). Finally, at line 5, the source code block should return a value to indicate success or the reason for failure.

```
(use-package :graph-dot)
(let
 <<declare-local-vars>>
 <<add-arcs-to-graph>>
 <<conditionally-plot-3>>
 <<return-value>>)
```

Listing S2. Noweb syntax for the Figure 3 source code block.

Two local variables, in addition to the two local variables created by the `:var` header arguments (see table args-graph-3-noweb), are needed for the Figure 3 source code block (Listing S3). The local variable `rejected` is declared to hold the first arc that fails to be added to the graph. It is initialized to `nil` implicitly. The local variable `graph` holds the directed graph (or digraph), and is initialized using the `(populate)` function declared in the `graph.lisp` library.

```
((rejected)
 (graph (populate (make-instance 'digraph))))
```

Listing S3. Declare local variables.

The source code to add arcs to the local variable `graph` makes use of the fact that, internally, the arcs from Table S3 are represented as a list (Listing S4). The Common Lisp `(dolist)` macro at Line 1 steps through the list of arcs. Lines 2–4 use the `add-edge` function from the `graph.lisp` library to add an arc to the directed graph. Line 5 checks to see if an arc has been rejected previously. If not, Line 6 checks whether addition of the arc has introduced a cycle, using the `cycles` function from the `graph.lisp` library. If a cycle has been introduced, then the arc is pushed on to the local variable `rejected`.

```
1 (dolist (arc arcs rejected)
2   (add-edge graph
3             (list (read-from-string (first arc))
4                   (read-from-string (second arc))))
5   (unless rejected
6     (and (cycles graph) (push arc rejected))))
```

Listing S4. Add arcs to a graph.

If no arc has been pushed on to the local variable `rejected` (Line 1), then the directed graph in the local variable `graph` is passed to the `to-dot-file` function from the `graph.lisp` library (Line 2), which is responsible for writing `dot` source code to the file named in the `out-file` header argument.

The keyword arguments to `(to-dot-file)`, `:attributes` and `:node-attrs`, take lists of key-value pairs that correspond to `dot` graph and node attributes. In Lines 3–7, the values for individual graph attributes are set. Lines 8-14 illustrate one of the joys of functional programming in Common Lisp. Here, an anonymous function `(lambda)` is applied to each node in `graph` and a node attribute is set according to the value of the function. Lines 11-13 check to see if the node label starts with the letter T and sets the shape of the node accordingly. This is a specific example of a more general pattern, where an anonymous function maps some characteristic of a graph to an attribute of a graph picture.

```
1  (unless rejected
2    (to-dot-file graph out-file
3                 :attributes (list (cons :bgcolor "white")
4                                   (cons :aspect 2.5)
5                                   (cons :fontname "Arial")
6                                   (cons :fontsize 10.0)
7                                   (cons :fontnames "gd"))
8                 :node-attrs (list
9                              (cons :shape
10                                   (lambda (n)
11                                     (if (string= "T" (string n)
12                                                  :start2 0
13                                                  :end2 1)
14                                         "box" "oval"))))))
```

Listing S5. Conditionally plot the Figure 3 graph picture.

Finally, the source code block returns a value that hopefully indicates something important about its performance (Listing S6). In this case, the value of `rejected` is `nil` if no cycles were found and the source code block tried to write the `dot` file. Otherwise, it reports the arc that introduced a cycle, so the illogical condition this indicates can be investigated and corrected.

```
rejected
```

Listing S6. Return a value from the source code block.

Source Code for the Figure 4 Graph Picture

The source code and data table for the Figure 3 and Figure 4 graph pictures are similar. The header arguments are identical (see table S4), and several of the `noweb` syntax code chunks can be re-used without modification.

Data table for Figure 4c

The data table for Figure 4 (table S5) has the same structure as the data table for Figure 3 (table S3), and the comments made there apply here, as well.

Table S5. Tabular representation of a directed graph with a cycle

Older	Younger
T1	A
A	T2
B	T1
T2	B

Structure of the Figure 4 Source Code Block

Listing S7 includes four noweb-style code chunks nested within a Common Lisp `(let)` special operator. Three of these, at Lines 1 (Listing S3), 2 (Listing S4), and 4 (Listing S6), refer to code chunks that were used to plot Figure 3. Only the code to plot Figure 4 is different.

```
1 (let
2  <<declare-local-vars>>
3  <<add-arcs-to-graph>>
4  <<conditionally-plot-4>>
5  <<return-value>>)
```

Listing S7. Noweb syntax for the Figure 4 source code block.

The source code to plot the Figure 4 graph picture reverses the condition used to plot Figure 3 by

changing the `(unless)` macro to a `(when)` macro on Line 1, but is otherwise the same (Listing S8). The object of Figure 4 is to illustrate a cycle, so the condition checks that a cycle is present before executing the plot code, and no plot code is generated unless a cycle is present.

```
1 (when rejected
2   (to-dot-file graph out-file
3                :attributes (list (cons :bgcolor "white")
4                                  (cons :aspect 2.5))
5                :node-attrs (list (cons :shape (lambda (n)
6                                                 (if (string= "T" (string n)
7                                                              :start2 0
8                                                              :end2 1)
9                                                     "box" "oval"))))))
```

Listing S8. Conditionally plot the Figure 4 graph picture.

Note that the expected return value when the source code block executes successfully is an arc, rather than `nil`.

Source Code for the Figure 5 Graph Picture

Figure 5 is designed to show, among other things, the ability to assign levels to the nodes of a directed graph and its relationship to relative chronology. Consequently, the primary difference in the source code has to do with level assignment and the representation of nodes to show levels. Two additional header arguments help the code accomplish this. The `:var` header argument `brewer-color` is used to set up the `dot` software to choose colors from a palette designed to work well with both web and print displays and a color-blind audience. The `:var` header argument `label-break` is used to control the color of the node label font.

Table S6. Arguments passed to the source code block in Listing S9.

Argument	Value
:var arcs	an Org mode table of nodes where the first two columns are “Older” and “Younger”
:var out-file	a string holding the name of the output file name with suffix, e.g. “file.dot”
:var brewer-color	a string that specifies a ColorBrewer palette
:var label-break	an integer that specifies the lowest level to label in white, rather than black
:dir	a path where the code will run and write its output

Data table for Figure 5

The data tables for Figure 5 (table S7) and Figure 3 (table S3) are similar, and the comments made there apply here, as well.

Table S7. Tabular representation of map with two trails

Older	Younger
T1	A
T1	B
D	T1
A	T2
T2	B
D	T2
T2	C

Structure of the Figure 5 Source Code Block

Listing S9 includes four noweb-style code chunks nested within a Common Lisp `(let)` special operator. Three of these, at Lines 1 (Listing S3), 2 (Listing S4), and 4 (Listing S6), refer to code chunks that were used to plot Figure 3. Only the code to plot Figure 5 is different.

```
1 (let
2  <<declare-local-vars>>
3  <<add-arcs-to-graph>>
4  <<conditionally-plot-5>>
5  <<return-value>>)
```

Listing S9. Noweb syntax for the Figure 5 source code block.

The source code to plot the Figure 5 graph picture (Listing S10) runs on the condition that no cycles have been found (Line 1). Line 2 declares a new local variable, `ls`, that is initialized with a call to the `levels` function in the `graph.lisp` library, which returns a hash table with the level assignment for each node. Lines 4 and 5 set the graph attributes as before. Several new elements are introduced when constructing the list for the `:node-attrs` keyword argument. Lines 7 and 8 configure the `dot` software to use the ColorBrewer palette specified by the `brewer-color` header argument. Lines 9 and 10 define an anonymous function to set a node's fill color according to its level assignment. Similarly, Lines 11–14 change the color of the label font according to the relationship between the node's level assignment and

the value given to the header argument `label-break`.

```
(unless rejected
  (let ((ls (levels graph)))
    (to-dot-file graph out-file
                 :attributes (list (cons :bgcolor "white")
                                   (cons :aspect 2.5))
                 :node-attrs (list
                              (cons :colorscheme
                                    (constantly brewer-color))
                              (cons :fillcolor (lambda (n)
                                                 (+ 1 (gethash n ls))))
                              (cons :fontcolor (lambda (n)
                                                 (if (<= label-break
                                                         (gethash n ls))
                                                     1 "\black")))
                              (cons  :shape (lambda (n)
                                              (if (string= "T" (string n)
                                                           :start2 0
                                                           :end2 1)
                                                  "box" "oval")))
                              (cons :style (constantly "filled"))))))
```

Listing S10. Conditionally plot the Figure 5 graph picture.

Source Code for the Figure 6 Graph Picture

Figure 6 is designed to illustrate the pictorial conventions used to illustrate subgraphs. The source code shows one way to identify subgraphs of a directed graph, and a general way to plot them.

Data table for Figure 6

The data table for Figure 6 (table S8) has the same structure as the data table for Figure 3 (table S3), and the comments made there apply here, as well.

Table S8. Tabular representation of connected components

Older	Younger
A	T2
T1	B
T2	B
T2	C
T1	D

Structure of the Figure 6 Source Code Block

Listing S11 includes four noweb-style code chunks nested within a Common Lisp `(let)` special operator. The header arguments for the source code block are the same as the previous figure (Table S6). Three of the code chunks, at Lines 1 (Listing S3), 2 (Listing S4), and 4 (Listing S6), refer to code chunks that were used to plot Figure 3. Only the code to plot Figure 5 is different.

```
1 (let
2  <<declare-local-vars>>
3  <<add-arcs-to-graph>>
4  <<conditionally-plot-6>>
5  <<return-value>>)
```

Listing S11. Noweb syntax for the Figure 6 source code block.

The source code to plot the Figure 6 graph picture (Listing S12) runs on the condition that no cycles have been found (Line 1). Line 2 declares a new local variable, `cc`, that is initialized with a call to the `connected-components` function in the `graph.lisp` library, which returns a list of lists, each of which contains the nodes of a unilaterally connected component. Line 3 declares and initializes the local variable `ls` as in Listing S10. Lines 5–9 introduce the `subgraphs` keyword argument. These lines step through the list held in the local variable `cc` and apply an anonymous function `(lambda)` to each of the list elements. The anonymous function calls the `(make-subgraph)` function in the `graph-dot.lisp` library and sets the attributes that distinguish subgraphs in the graph picture. Line 7 sets the color attribute so the subgraphs are indicated by a gray box and Line 8 declines the opportunity to label each subgraph. The rest of the source code block is identical to Listing S10.

```
(unless rejected
  (let ((cc (connected-components graph :type :unilateral))
        (ls (levels graph)))
    (to-dot-file graph out-file
                 :subgraphs (mapcar (lambda (x)
                                      (make-subgraph
                                       :attributes '(("color" . "gray")
                                                     ("label" . "" ))
                                       :node-list x)) cc)
                 :attributes (list (cons :bgcolor "white")
                                   (cons :aspect 2.5))
                 :node-attrs (list
                              (cons :shape (lambda (n)
                                             (if (string= "T" (string n)
                                                          :start2 0
                                                          :end2 1)
                                                 "box" "oval")))
                              (cons :style (constantly "filled"))
                              (cons :colorscheme
                                    (constantly brewer-color))
                              (cons :fillcolor (lambda (n)
                                                 (+ 1 (gethash n ls))))
                              (cons :fontcolor (lambda (n)
                                                 (if (<= label-break
                                                         (gethash n ls))
                                                     1 "\black")))))))
```

Listing S12. Conditionally plot the Figure 6 graph picture.

Investigation of Lapakahi Detailed Study Area

This section presents the data used to model relative chronological relationships in the Lapakahi detailed study area and the source code used to plot a graph picture of the model.

Relative Chronological Relationships at Lapakahi

Table S9 holds information about the relative chronological relationships of agricultural walls and trails in the Lapakahi detailed study area. This information was used to model the structure and growth of the field system. The first two columns hold the labels of the `Older` and `Younger` features. The third column, `Source`, indicates whether the observation that established the relative chronological relationship was made on the plane-table map of the detailed study area (see the file `lapakahi-dsa-map.png`) or on Google Earth imagery. This column also includes a date stamp when the information was checked prior to finalizing the model for analysis and publication. Labels assigned to the agricultural walls and trails are shown on the map `lapakahi-dsa-final.kmz`.

Table S9. Chronological relationships in the detailed study area at Lapakahi.

Older	Younger	Source
IIIb	W053	Google Earth *[2013-12-03 Tue]*
IIIb	W064	Google Earth *[2013-12-03 Tue]*
IIIb	W066	Google Earth *[2013-12-03 Tue]*
IIIb	W069	Google Earth *[2013-12-03 Tue]*
IIIb	W090	Google Earth *[2013-12-03 Tue]*
IIIb	W099	Google Earth *[2013-12-03 Tue]*
IIIb	W117	Google Earth *[2013-12-03 Tue]*
IIIb	W126	Google Earth *[2013-12-03 Tue]*
IIIc	W038	map *[2013-12-02 Mon]*
IIIc	W040	map *[2013-12-02 Mon]*
IIIc	W041	map *[2013-12-02 Mon]*
IIIc	W042	map *[2013-12-02 Mon]*
IIIc	W043	map *[2013-12-02 Mon]*
IIIc	W044	map *[2013-12-02 Mon]*
IIIc	W045	map *[2013-12-02 Mon]*
IIIc	W046	map *[2013-12-02 Mon]*
IIIc	W048	map *[2013-12-02 Mon]*
IIIc	W053	map *[2013-12-02 Mon]*
IIIc	W054	map *[2013-12-02 Mon]*
IIIc	W055	map *[2013-12-02 Mon]*
IIIc	W056	map *[2013-12-02 Mon]*
IIIc	W057	map *[2013-12-02 Mon]*

continued on next page . . .

. . . Table S9 continued from previous page

Older	Younger	Source
IIIc	W060	map *[2013-12-02 Mon]*
IIIc	W061	map *[2013-12-02 Mon]*
IIIc	W063	map *[2013-12-02 Mon]*
IIIc	W064	map *[2013-12-02 Mon]*
IIIc	W065	map *[2013-12-02 Mon]*
IIIc	W066	map *[2013-12-02 Mon]*
IIIc	W067	map *[2013-12-02 Mon]*
IIIc	W069	map *[2013-12-02 Mon]*
IIIc	W070	map *[2013-12-02 Mon]*
IIIc	W071	map *[2013-12-02 Mon]*
IIIc	W072	map *[2013-12-02 Mon]*
IIIc	W073	map *[2013-12-02 Mon]*
IIIc	W075	map *[2013-12-02 Mon]*
IIIc	W076	map *[2013-12-02 Mon]*
IIIc	W082	map *[2013-12-02 Mon]*
IIIc	W083	map *[2013-12-02 Mon]*
IIIc	W085	map *[2013-12-02 Mon]*
IIIc	W088	map *[2013-12-02 Mon]*
IIIc	W089	map *[2013-12-02 Mon]*
IIIc	W090	map *[2013-12-02 Mon]*
IIIc	W091	map *[2013-12-02 Mon]*
IIIc	W092	map *[2013-12-02 Mon]*
IIIc	W093	map *[2013-12-02 Mon]*
IIIc	W094	map *[2013-12-02 Mon]*
IIIc	W095	map *[2013-12-02 Mon]*
IIIc	W096	map *[2013-12-02 Mon]*
IIIc	W097	map *[2013-12-02 Mon]*
IIIc	W098	map *[2013-12-02 Mon]*
IIIc	W099	map *[2013-12-02 Mon]*
IIIc	W100	map *[2013-12-02 Mon]*
IIIc	W101	map *[2013-12-02 Mon]*
IIIc	W102	map *[2013-12-02 Mon]*
IIIc	W103	map *[2013-12-02 Mon]*
IIIc	W105	map *[2013-12-02 Mon]*

continued on next page . . .

. . . Table S9 continued from previous page

Older	Younger	Source
IIIc	W106	map *[2013-12-02 Mon]*
IIIc	W108	map *[2013-12-02 Mon]*
IIIc	W109	map *[2013-12-02 Mon]*
IIIc	W110	map *[2013-12-02 Mon]*
IIIc	W112	map *[2013-12-02 Mon]*
IIIc	W113	map *[2013-12-02 Mon]*
IIIc	W114	map *[2013-12-02 Mon]*
IIIc	W115	map *[2013-12-02 Mon]*
IIIc	W117	map *[2013-12-02 Mon]*
IIIc	W118	map *[2013-12-02 Mon]*
IIIc	W119	map *[2013-12-02 Mon]*
IIIc	W123	map *[2013-12-02 Mon]*
IIIc	W124	map *[2013-12-02 Mon]*
IIIc	W125	map *[2013-12-02 Mon]*
IIIc	W126	map *[2013-12-02 Mon]*
IIIc	W127	map *[2013-12-02 Mon]*
IIIc	W128	map *[2013-12-02 Mon]*
IIIc	W129	map *[2013-12-02 Mon]*
IIIc	W132	map *[2013-12-02 Mon]*
IIIc	W133	map *[2013-12-02 Mon]*
IIIc	W134	map *[2013-12-02 Mon]*
IIIc	W137	map *[2013-12-02 Mon]*
IIIc	W139	map *[2013-12-02 Mon]*
IIIc	W140	map *[2013-12-02 Mon]*
IIIc	W142	map *[2013-12-02 Mon]*
IIIc	W145	map *[2013-12-02 Mon]*
IIIc	W147	map *[2013-12-02 Mon]*
IIIc	W149	map *[2013-12-02 Mon]*
IIIc	W151	map *[2013-12-02 Mon]*
IIIc	W152	map *[2013-12-02 Mon]*
IIIc	W153	map *[2013-12-02 Mon]*
IIIc	W154	map *[2013-12-02 Mon]*
IIIc	W158	map *[2013-12-02 Mon]*
IIIc	W162	map *[2013-12-02 Mon]*

continued on next page . . .

. . . Table S9 continued from previous page

Older	Younger	Source
IIIc	W161	map *[2013-12-02 Mon]*
IIIc	W163	map *[2013-12-02 Mon]*
IIIc	W164	map *[2013-12-02 Mon]*
IIIc	W167	map *[2013-12-02 Mon]*
IIIc	W168	map *[2013-12-02 Mon]*
IIIc	W170	map *[2013-12-02 Mon]*
IIIc	W171	map *[2013-12-02 Mon]*
IIIc	W172	map *[2013-12-02 Mon]*
IIIc	W174	map *[2013-12-02 Mon]*
IIIc	W175	map *[2013-12-02 Mon]*
IIIc	W176	map *[2013-12-02 Mon]*
IIIc	W177	map *[2013-12-02 Mon]*
IIIc	W179	map *[2013-12-02 Mon]*
IIIc	W180	map *[2013-12-02 Mon]*
IIIc	W182	map *[2013-12-02 Mon]*
IIIc	W183	map *[2013-12-02 Mon]*
IIIc	W189	map *[2013-12-02 Mon]*
IIIc	W191	map *[2013-12-02 Mon]*
IIIc	W192	map *[2013-12-02 Mon]*
IIIc	W194	map *[2013-12-02 Mon]*
IIIc	W195	map *[2013-12-02 Mon]*
IIIc	W196	map *[2013-12-02 Mon]*
IIIc	W197	map *[2013-12-02 Mon]*
IIIc	W198	map *[2013-12-02 Mon]*
IIIc	W199	map *[2013-12-02 Mon]*
IIIc	W200	map *[2013-12-02 Mon]*
IIIc	W201	map *[2013-12-02 Mon]*
IIIc	W203	map *[2013-12-02 Mon]*
IIIc	W204	map *[2013-12-02 Mon]*
IIIc	W205	map *[2013-12-02 Mon]*
IIIc	W206	map *[2013-12-02 Mon]*
IIIc	W207	map *[2013-12-02 Mon]*
IIIc	W208	map *[2013-12-02 Mon]*
IIIc	W209	map *[2013-12-02 Mon]*

continued on next page . . .

. . . Table S9 continued from previous page

Older	Younger	Source
IIIc	W210	map *[2013-12-02 Mon]*
IIIc	W211	map *[2013-12-02 Mon]*
IIIc	W215	map *[2013-12-02 Mon]*
IIIc	W216	map *[2013-12-02 Mon]*
IIIc	W217	map *[2013-12-02 Mon]*
IIIc	W218	map *[2013-12-02 Mon]*
IIIc	W219	map *[2013-12-02 Mon]*
IIIc	W220	map *[2013-12-02 Mon]*
IIIc	W221	map *[2013-12-02 Mon]*
IIIc	W222	map *[2013-12-02 Mon]*
IIIc	W223	map *[2013-12-02 Mon]*
IIIc	W225	map *[2013-12-02 Mon]*
IIIc	W239	map *[2013-12-16 Mon]*
IVd	W051	map *[2013-12-02 Mon]*
IVd	W059	map *[2013-12-02 Mon]*
IVe	W052	map *[2013-12-02 Mon]*
IVe	W078	map *[2013-12-02 Mon]*
IVe	W079	map *[2013-12-02 Mon]*
IVe	W081	map *[2013-12-02 Mon]*
IVe	W083	map *[2013-12-02 Mon]*
IVe	W084	map *[2013-12-02 Mon]*
IVe	W087	map *[2013-12-02 Mon]*
IVe	W110	map *[2013-12-02 Mon]*
IVe	W111	map *[2013-12-02 Mon]*
IVe	W142	map *[2013-12-02 Mon]*
IVe	W143	map *[2013-12-02 Mon]*
IVe	W085	map *[2013-12-16 Mon]*
IVe	W237	map *[2013-12-16 Mon]*
IVe	W076	map *[2013-12-16 Mon]*
IVe	W239	map *[2013-12-16 Mon]*
IVg	W189	map *[2013-12-02 Mon]*
IVh	W173	map *[2013-12-02 Mon]*
IVh	W235	map *[2013-12-02 Mon]*
IVh	W181	map *[2013-12-02 Mon]*

continued on next page . . .

. . . Table S9 continued from previous page

Older	Younger	Source
IVh	W184	map *[2013-12-02 Mon]*
IVi	W200	Google Earth *[2013-12-03 Tue]*
Va	W035	map *[2013-12-02 Mon]*
Va	W038	map *[2013-12-02 Mon]*
Va	W044	map *[2013-12-02 Mon]*
Va	W061	map *[2013-12-02 Mon]*
Va	W067	map *[2013-12-02 Mon]*
Va	W046	map *[2013-12-03 Tue]*
VIa	W237	map *[2013-12-16 Mon]*
W018	IVa	Google Earth *[2013-12-03 Tue]*
W019	IVa	Google Earth *[2013-12-03 Tue]*
W020	IVa	Google Earth *[2013-12-03 Tue]*
W021	IVa	Google Earth *[2013-12-03 Tue]*
W022	IVa	Google Earth *[2013-12-03 Tue]*
W023	IVa	Google Earth *[2013-12-03 Tue]*
W027	IVb	Google Earth *[2013-12-03 Tue]*
W028	IVb	Google Earth *[2013-12-03 Tue]*
W029	IVb	Google Earth *[2013-12-03 Tue]*
W032	IVc	map *[2013-12-02 Mon]*
W032	IVb	Google Earth *[2013-12-03 Tue]*
W034	IVb	Google Earth *[2013-12-03 Tue]*
W036	IVb	Google Earth *[2013-12-03 Tue]*
W037	Va	map *[2013-12-02 Mon]*
W039	Va	map *[2013-12-02 Mon]*
W039	IVc	map *[2013-12-02 Mon]*
W042	Va	map *[2013-12-02 Mon]*
W042	IVc	map *[2013-12-02 Mon]*
W043	IIIb	Google Earth *[2013-12-03 Tue]*
W045	IIIb	Google Earth *[2013-12-03 Tue]*
W047	IVe	map *[2013-12-02 Mon]*
W048	IIIb	Google Earth *[2013-12-03 Tue]*
W049	IIIc	map *[2013-12-02 Mon]*
W055	IIIb	Google Earth *[2013-12-03 Tue]*
W057	IIIb	Google Earth *[2013-12-03 Tue]*

continued on next page . . .

... Table S9 continued from previous page

Older	Younger	Source
W058	IIIc	map *[2013-12-02 Mon]*
W058	IIIb	Google Earth *[2013-12-03 Tue]*
W060	IIIb	Google Earth *[2013-12-03 Tue]*
W062	IVd	map *[2013-12-02 Mon]*
W062	IVe	map *[2013-12-02 Mon]*
W063	IIIb	Google Earth *[2013-12-03 Tue]*
W065	Va	map *[2013-12-02 Mon]*
W068	IVd	map *[2013-12-02 Mon]*
W070	IIIb	Google Earth *[2013-12-03 Tue]*
W071	Va	map *[2013-12-02 Mon]*
W072	IIIb	Google Earth *[2013-12-03 Tue]*
W075	IIIb	Google Earth *[2013-12-03 Tue]*
W077	IIIc	map *[2013-12-02 Mon]*
W077	IIIb	Google Earth *[2013-12-03 Tue]*
W078	VIa	map *[2013-12-04 Wed]*
W079	VIa	map *[2013-12-04 Wed]*
W080	IIIc	map *[2013-12-02 Mon]*
W080	IVe	map *[2013-12-02 Mon]*
W080	VIa	map *[2013-12-02 Mon]*
W080	IIIb	Google Earth *[2013-12-03 Tue]*
W082	IIIb	Google Earth *[2013-12-03 Tue]*
W084	VIa	map *[2013-12-02 Mon]*
W088	IVe	map *[2013-12-02 Mon]*
W088	VIa	map *[2013-12-03 Tue]*
W089	IIIb	Google Earth *[2013-12-03 Tue]*
W091	IVe	map *[2013-12-02 Mon]*
W091	VIa	map *[2013-12-02 Mon]*
W092	IIIb	Google Earth *[2013-12-03 Tue]*
W093	IVe	map *[2013-12-02 Mon]*
W094	IIIb	Google Earth *[2013-12-03 Tue]*
W095	IVe	map *[2013-12-02 Mon]*
W096	IIIb	Google Earth *[2013-12-03 Tue]*
W097	IIIb	Google Earth *[2013-12-03 Tue]*
W098	IVe	map *[2013-12-02 Mon]*

continued on next page ...

. . . Table S9 continued from previous page

Older	Younger	Source
W100	IIIb	Google Earth *[2013-12-03 Tue]*
W102	IVe	map *[2013-12-02 Mon]*
W102	IVf	Google Earth *[2013-12-03 Tue]*
W104	IIIc	map *[2013-12-02 Mon]*
W104	IVe	map *[2013-12-02 Mon]*
W107	IVe	map *[2013-12-02 Mon]*
W108	IVe	map *[2013-12-02 Mon]*
W109	IIIb	Google Earth *[2013-12-03 Tue]*
W113	IVe	map *[2013-12-02 Mon]*
W113	IVf	Google Earth *[2013-12-03 Tue]*
W114	IIIb	Google Earth *[2013-12-03 Tue]*
W116	IVf	Google Earth *[2013-12-03 Tue]*
W118	IIIb	Google Earth *[2013-12-03 Tue]*
W119	IVe	map *[2013-12-02 Mon]*
W120	IIIc	map *[2013-12-02 Mon]*
W120	IVe	map *[2013-12-02 Mon]*
W120	IVf	Google Earth *[2013-12-03 Tue]*
W120	IIIb	Google Earth *[2013-12-03 Tue]*
W121	IVf	Google Earth *[2013-12-03 Tue]*
W122	IIIc	map *[2013-12-02 Mon]*
W122	IVe	map *[2013-12-02 Mon]*
W122	IVf	Google Earth *[2013-12-03 Tue]*
W122	IIIb	Google Earth *[2013-12-03 Tue]*
W123	IIIb	Google Earth *[2013-12-03 Tue]*
W124	IVe	map *[2013-12-02 Mon]*
W124	IVf	Google Earth *[2013-12-03 Tue]*
W125	IIIb	Google Earth *[2013-12-03 Tue]*
W127	IIIb	Google Earth *[2013-12-03 Tue]*
W128	IVe	map *[2013-12-02 Mon]*
W128	IVf	Google Earth *[2013-12-03 Tue]*
W129	IIIb	Google Earth *[2013-12-03 Tue]*
W130	IVe	map *[2013-12-02 Mon]*
W132	IIIb	Google Earth *[2013-12-03 Tue]*
W133	IVe	map *[2013-12-02 Mon]*

continued on next page . . .

. . . Table S9 continued from previous page

Older	Younger	Source
W134	IIIb	Google Earth *[2013-12-03 Tue]*
W135	IIIc	map *[2013-12-02 Mon]*
W135	IVe	map *[2013-12-02 Mon]*
W135	IIIb	Google Earth *[2013-12-03 Tue]*
W137	IVe	map *[2013-12-02 Mon]*
W137	IVf	Google Earth *[2013-12-03 Tue]*
W139	IIIb	Google Earth *[2013-12-03 Tue]*
W140	IVe	map *[2013-12-02 Mon]*
W140	IVf	Google Earth *[2013-12-03 Tue]*
W144	IVf	Google Earth *[2013-12-03 Tue]*
W147	IVe	map *[2013-12-02 Mon]*
W149	IVe	map *[2013-12-02 Mon]*
W151	IVe	map *[2013-12-02 Mon]*
W155	IIIc	map *[2013-12-02 Mon]*
W155	IVe	map *[2013-12-02 Mon]*
W157	IIIc	map *[2013-12-02 Mon]*
W157	IVe	map *[2013-12-02 Mon]*
W158	IIIb	Google Earth *[2013-12-03 Tue]*
W159	IIIc	map *[2013-12-02 Mon]*
W159	IIIb	Google Earth *[2013-12-03 Tue]*
W160	IIIc	map *[2013-12-02 Mon]*
W160	IVg	map *[2013-12-02 Mon]*
W160	IVh	map *[2013-12-02 Mon]*
W160	IIIb	Google Earth *[2013-12-03 Tue]*
W162	IIIb	Google Earth *[2013-12-03 Tue]*
W163	IVg	map *[2013-12-02 Mon]*
W163	IVg	map *[2013-12-02 Mon]*
W165	IIIc	map *[2013-12-02 Mon]*
W165	IVg	map *[2013-12-02 Mon]*
W165	IVh	map *[2013-12-02 Mon]*
W166	IVh	map *[2013-12-02 Mon]*
W166	IVf	Google Earth *[2013-12-03 Tue]*
W168	IVh	map *[2013-12-02 Mon]*
W168	IVg	map *[2013-12-02 Mon]*

continued on next page . . .

. . . Table S9 continued from previous page

Older	Younger	Source
W168	IVg	map *[2013-12-02 Mon]*
W168	IVf	Google Earth *[2013-12-03 Tue]*
W169	IVh	map *[2013-12-02 Mon]*
W171	IVh	map *[2013-12-02 Mon]*
W171	IVg	map *[2013-12-02 Mon]*
W171	IVg	map *[2013-12-02 Mon]*
W171	IVf	Google Earth *[2013-12-03 Tue]*
W172	IIIb	Google Earth *[2013-12-03 Tue]*
W174	IVh	map *[2013-12-02 Mon]*
W174	IVg	map *[2013-12-02 Mon]*
W176	IIIb	Google Earth *[2013-12-03 Tue]*
W177	IVh	map *[2013-12-02 Mon]*
W177	IVg	map *[2013-12-02 Mon]*
W177	IVf	Google Earth *[2013-12-03 Tue]*
W178	IVh	map *[2013-12-02 Mon]*
W179	IIIb	Google Earth *[2013-12-03 Tue]*
W180	IVg	map *[2013-12-02 Mon]*
W182	IIIb	Google Earth *[2013-12-03 Tue]*
W183	IVg	map *[2013-12-02 Mon]*
W186	IIIc	map *[2013-12-02 Mon]*
W186	IVg	map *[2013-12-02 Mon]*
W186	IIIb	Google Earth *[2013-12-03 Tue]*
W187	IIIc	map *[2013-12-02 Mon]*
W187	IVg	map *[2013-12-02 Mon]*
W187	IIIb	Google Earth *[2013-12-03 Tue]*
W188	IIIc	map *[2013-12-02 Mon]*
W188	IVg	map *[2013-12-02 Mon]*
W188	IIIb	Google Earth *[2013-12-03 Tue]*
W190	IIIc	map *[2013-12-02 Mon]*
W190	IVk	map *[2013-12-02 Mon]*
W190	IVj	map *[2013-12-02 Mon]*
W190	IIIb	Google Earth *[2013-12-03 Tue]*
W191	IVk	map *[2013-12-02 Mon]*
W191	IVj	map *[2013-12-02 Mon]*

continued on next page . . .

. . . Table S9 continued from previous page

Older	Younger	Source
W193	IVk	map *[2013-12-02 Mon]*
W193	IVj	map *[2013-12-02 Mon]*
W194	IVk	map *[2013-12-02 Mon]*
W194	IVj	map *[2013-12-02 Mon]*
W201	IVi	Google Earth *[2013-12-03 Tue]*
W212	IVi	Google Earth *[2013-12-03 Tue]*
W213	IIIc	map *[2013-12-02 Mon]*
W214	IIIc	map *[2013-12-02 Mon]*
W224	IIIc	map *[2013-12-02 Mon]*
W236	IVh	map *[2013-12-02 Mon]*

Plot a Graph of the Lapakahi Detailed Study Area

Listing S13 includes four noweb-style code chunks nested within a Common Lisp `(let)` special operator. In addition to the header arguments used for other source code blocks, the argument `:var components` is used to toggle marking of subgraphs (Table S10). Three of these, at Lines 1 (Listing S3), 2 (Listing S4), and 4 (Listing S6), refer to code chunks that were used to plot Figure 3. Only the code to plot the directed graph of the Lapakahi detailed study area is different.

Table S10. Arguments passed to the source code block in Listing S13.

Argument	Value
:var arcs	an Org mode table of nodes where the first two columns are “Older” and “Younger”
:var out-file	a string holding the name of the output file name with suffix, e.g. “file.dot”
:var brewer-color	a string that specifies a ColorBrewer palette
:var label-break	an integer that specifies the lowest level to label in white, rather than black
:var components	an Emacs-lisp function that returns a boolean value
:dir	a path where the code will run and write its output

```
1 (let
2  <<declare-local-vars>>
3  <<add-arcs-to-graph>>
4  <<conditionally-plot-lapakahi>>
5  <<return-value>>)
```

Listing S13. Noweb syntax for the Lapakahi source code block.

There are two steps needed to plot the directed graph of the Lapakahi detailed study area. First, because the trails were treated as segments, they need to be merged to construct archaeologically meaningful unilaterally connected subgraphs (Line 3). Once this is accomplished, then the `dot` code can be generated in the usual way (Line 4). Because the subgraphs are held in a local variable, Lines 3 and 4 are nested within a `(let)` special operator. All of this is nested in an `(unless)` macro so the code only executes if no cycles were encountered.

```
1 (unless rejected
2   (let
3       <<lapakahi-subgraphs>>
4       <<plot-lapakahi-graph>>
5     ))
```

Listing S14. Conditionally plot the Lapakahi graph picture.

The source code block to identify unilaterally connected subgraphs at Lapakahi (Listing S15) calls the `connected-component` function from the `graph.lisp` library, which returns a list of all the nodes reachable from a given node. The nodes are five trail segments that, together, reach all of the level 2 and higher agricultural walls in the detailed study area. These include four early trail segments—IVA, IVD, IVB, and IIIC—along with trail segment VIA, which appears to be late but is not connected to another trail.

```
((p-levels (levels graph))
 (p-comps (list (connected-component
                 graph
                 (read-from-string "IVA")
                 :type :unilateral)
                (connected-component
                 graph
                 (read-from-string "IVD")
                 :type :unilateral)
                (connected-component
                 graph
                 (read-from-string "IVB")
                 :type :unilateral)
                (connected-component
                 graph
                 (read-from-string "VIA")
                 :type :unilateral)
                (connected-component
                 graph
                 (read-from-string "IIIC")
                 :type :unilateral))))
```

Listing S15. Identify unilaterally connected subgraphs at Lapakahi.

The source code block to plot the Lapakahi detailed study area graph picture (Listing S16) is similar to the source code block used to plot Figure 6 (Listing S12), except that, for aesthetic reasons, it sets an edge attribute to render arcs in gray, instead of black.

```
(to-dot-file graph out-file
                        :attributes (list (cons :bgcolor "gray97")
                                          (cons :aspect 2.5))
                        :edge-attrs (list (cons :color (constantly "grey")))
                        :node-attrs (list
                                     (cons :colorscheme (constantly brewer-color))
                                     (cons :fillcolor (lambda (n)
                                                        (+ 1 (gethash n p-levels))))
                                     (cons :fontcolor (lambda (n)
                                                        (if (<= label-break
                                                                (gethash n p-levels))
                                                            1 "\black")))
                                     (cons  :shape (lambda (n)
                                                     (if (string= "W" (string n)
                                                                  :start2 0
                                                                  :end2 1)
                                                         "oval" "box")))
                                     (cons  :style (constantly "filled")))
                        :subgraphs (and components
                                        (mapcar (lambda (x)
                                                  (graph-dot:make-subgraph
                                                   :attributes '(("color" . "red")
                                                                 ("label" . "" ))
                                                   :node-list x)) p-comps)))
```

Listing S16. Plot the Lapakahi detailed study area graph picture.

Investigation of Kahua 1 and Pāhinahina Detailed Study Area

This section presents the data used to model relative chronological relationships in the Kahua 1 and Pāhinahina detailed study area and the source code used to plot a graph picture of the model.

Relative Chronological Relationships at Kahua 1 and Pāhinahina

Table S11 holds information about the relative chronological relationships of features in the Kahua 1 and Pāhinahina detailed study area. This information was used to model the structure and growth of the field

system. The first two columns hold the labels of the `Older` and `Younger` features. The third column, `Source`, indicates whether the observation that established the relative chronological relationship was made on the field system map [7] or with information provided by Thegn Ladefoged in email correspondence. This column also includes a date stamp when the information was checked prior to finalizing the model for analysis and publication. Labels assigned to the agricultural walls and trails are shown on the map `kahua-dsa-final.kmz`.

Table S11. Chronological relationships in the detailed study area at Kahua 1 and Pāhinahina.

Older	Younger	Source
T001	W001	map *[2013-11-04 Mon]*
T001	W005	map *[2013-11-04 Mon]*
T001	W006	map *[2013-11-04 Mon]*
T001	W008	map *[2013-11-04 Mon]*
T001	W009	map *[2013-11-04 Mon]*
T001	W022	map *[2013-11-04 Mon]*
T001	W025	map *[2013-11-04 Mon]*
T001	W026	map *[2013-11-04 Mon]*
T001	W027	map *[2013-11-04 Mon]*
T001	W028	map *[2013-11-04 Mon]*
T001	W029	map *[2013-11-04 Mon]*
T001	W030	map *[2013-11-04 Mon]*
T001	W031	map *[2013-11-04 Mon]*
T001	W032	map *[2013-11-04 Mon]*
T001	W033	map *[2013-11-04 Mon]*
T001	W034	map *[2013-11-04 Mon]*
T001	W035	map *[2013-11-04 Mon]*
T002	W002	map *[2013-11-04 Mon]*
T002	W011	map *[2013-11-04 Mon]*
T002	W012	map *[2013-11-04 Mon]*
T002	W013	map *[2013-11-04 Mon]*
T002	W014	map *[2013-11-04 Mon]*
T002	W015	map *[2013-11-04 Mon]*
T002	W016	map *[2013-11-04 Mon]*
T002	W017	map *[2013-11-04 Mon]*

continued on next page . . .

. . . Table S11 continued from previous page

Older	Younger	Source
T002	W018	map *[2013-11-04 Mon]*
T002	W027	map *[2013-11-04 Mon]*
T002	W028	map *[2013-11-04 Mon]*
T002	W033	map *[2013-11-04 Mon]*
T002	W036	map *[2013-11-04 Mon]*
T002	W037	map *[2013-11-04 Mon]*
T002	W040	map *[2013-11-04 Mon]*
T002	W041	map *[2013-11-04 Mon]*
T002	W043	map *[2013-11-04 Mon]*
T002	W044	map *[2013-11-04 Mon]*
T002	W046	map *[2013-11-04 Mon]*
T002	W048	map *[2013-11-04 Mon]*
T002	W049	map *[2013-11-04 Mon]*
T002	W050	map *[2013-11-04 Mon]*
T002	W103	map *[2013-11-04 Mon]*
T002	W104	map *[2013-11-04 Mon]*
T002	W105	map *[2013-11-04 Mon]*
T002	W106	map *[2013-11-04 Mon]*
T003	W036	map *[2013-11-04 Mon]*
T003	W037	map *[2013-11-04 Mon]*
T003	W038	map *[2013-11-04 Mon]*
T003	W039	map *[2013-11-04 Mon]*
T003	W042	map *[2013-11-04 Mon]*
T003	W043	map *[2013-11-04 Mon]*
T003	W044	map *[2013-11-04 Mon]*
T003	W045	map *[2013-11-04 Mon]*
T003	W046	map *[2013-11-04 Mon]*
T003	W047	map *[2013-11-04 Mon]*
T003	W053	map *[2013-11-04 Mon]*
T003	W054	map *[2013-11-04 Mon]*
T003	W055	map *[2013-11-04 Mon]*
T003	W056	map *[2013-11-04 Mon]*
T004	W053	map *[2013-11-04 Mon]*
T004	W058	map *[2013-11-04 Mon]*

continued on next page . . .

. . . Table S11 continued from previous page

Older	Younger	Source
T004	W060	map *[2013-11-04 Mon]*
T004	W067	map *[2013-11-04 Mon]*
T004	W069	map *[2013-11-04 Mon]*
T004	W072	map *[2013-11-04 Mon]*
T004	W073	map *[2013-11-04 Mon]*
T005	W058	map *[2013-11-04 Mon]*
T005	W064	map *[2013-11-04 Mon]*
T005	W067	map *[2013-11-04 Mon]*
T005	W068	map *[2013-11-04 Mon]*
T005	W069	map *[2013-11-04 Mon]*
T005	W070	map *[2013-11-04 Mon]*
T005	W071	map *[2013-11-04 Mon]*
T006	W057	map *[2013-11-04 Mon]*
T006	W060	email *[2013-03-25 Mon]*
T006	W062	map *[2013-11-04 Mon]*
T006	W063	map *[2013-11-04 Mon]*
T006	W066	map *[2013-11-04 Mon]*
T006	W071	map *[2013-11-04 Mon]*
T006	W074	map *[2013-11-04 Mon]*
T006	W075	map *[2013-11-04 Mon]*
T006	W081	map *[2013-11-04 Mon]*
T006	W082	map *[2013-11-04 Mon]*
T006	W083	map *[2013-11-04 Mon]*
T006	W088	map *[2013-11-04 Mon]*
T006	W089	map *[2013-11-04 Mon]*
T006	W090	map *[2013-11-04 Mon]*
T006	W091	map *[2013-11-04 Mon]*
T006	W093	map *[2013-11-04 Mon]*
T006	W097	map *[2013-11-04 Mon]*
T006	W098	map *[2013-11-04 Mon]*
T006	W099	map *[2013-11-04 Mon]*
T006	W101	map *[2013-11-04 Mon]*
T006	W102	map *[2013-11-04 Mon]*
T006	W108	map *[2013-11-04 Mon]*

continued on next page . . .

. . . Table S11 continued from previous page

Older	Younger	Source
T006	W111	map *[2013-11-04 Mon]*
W002	T001	map *[2013-11-04 Mon]*
W003	T001	map *[2013-11-04 Mon]*
W004	T001	map *[2013-11-04 Mon]*
W007	T001	map *[2013-11-04 Mon]*
W007	T002	map *[2013-11-04 Mon]*
W010	T001	map *[2013-11-04 Mon]*
W011	T001	map *[2013-11-04 Mon]*
W012	T001	map *[2013-11-04 Mon]*
W013	T001	map *[2013-11-04 Mon]*
W014	T001	map *[2013-11-04 Mon]*
W015	T001	map *[2013-11-04 Mon]*
W016	T001	map *[2013-11-04 Mon]*
W017	T001	map *[2013-11-04 Mon]*
W018	T001	map *[2013-11-04 Mon]*
W019	T001	map *[2013-11-04 Mon]*
W019	T002	map *[2013-11-04 Mon]*
W019	T003	map *[2013-11-04 Mon]*
W020	T001	map *[2013-11-04 Mon]*
W020	T002	map *[2013-11-04 Mon]*
W020	T003	map *[2013-11-04 Mon]*
W021	T001	map *[2013-11-04 Mon]*
W021	T002	map *[2013-11-04 Mon]*
W021	T003	map *[2013-11-04 Mon]*
W023	T001	map *[2013-11-04 Mon]*
W024	T001	map *[2013-11-04 Mon]*
W048	T003	map *[2013-11-04 Mon]*
W049	T003	map *[2013-11-04 Mon]*
W050	T003	map *[2013-11-04 Mon]*
W057	T005	map *[2013-11-04 Mon]*
W059	T005	map *[2013-11-04 Mon]*
W060	T005	map *[2013-11-04 Mon]*
W063	T004	map *[2013-11-04 Mon]*
W063	T005	map *[2013-11-04 Mon]*

continued on next page . . .

. . . Table S11 continued from previous page

Older	Younger	Source
W066	T005	map *[2013-11-04 Mon]*
W072	T005	map *[2013-11-04 Mon]*
W075	T005	map *[2013-11-04 Mon]*
W076	T005	map *[2013-11-04 Mon]*
W076	T006	map *[2013-11-04 Mon]*
W077	T005	map *[2013-11-04 Mon]*
W077	T006	map *[2013-11-04 Mon]*
W078	T005	map *[2013-11-04 Mon]*
W078	T006	map *[2013-11-04 Mon]*
W079	T005	map *[2013-11-04 Mon]*
W080	T006	map *[2013-11-04 Mon]*
W084	T005	map *[2013-11-04 Mon]*
W084	T006	map *[2013-11-04 Mon]*
W084	T006	map *[2013-11-04 Mon]*
W084	T007	map *[2013-11-04 Mon]*
W085	T005	map *[2013-11-04 Mon]*
W085	T006	map *[2013-11-04 Mon]*
W087	T006	map *[2013-11-04 Mon]*

Plot a Graph of the Kahua 1 and Pāhinahina Study Area

Listing S17 includes four noweb-style code chunks nested within a Common Lisp `(let)` special operator. The header arguments are described in Table S10. Three of these, at Lines 1 (Listing S3), 2 (Listing S4), and 4 (Listing S6), refer to code chunks that were used to plot Figure 3. Only the code to plot the directed graph of the Kahua 1 and Pāhinahina detailed study area (Line 4) is different.

```
1 (let
2  <<declare-local-vars>>
3  <<add-arcs-to-graph>>
4  <<conditionally-plot-kahua>>
5  <<return-value>>)
```

Listing S17. Noweb syntax for the Kahua 1 and Pāhinahina source code block.

There are two steps needed to plot the directed graph of the Kahua 1 and Pāhinahina detailed study area. First, subgraphs of the nodes reachable from the earliest trails are defined (Line 3). Once this is accomplished, then the `dot` code can be generated in the usual way (Line 4). Because the subgraphs are held in a local variable, Lines 3 and 4 are nested within a `(let)` special operator. All of this is nested in an `(unless)` macro so the code only executes if no cycles were encountered.

```
1 (unless rejected
2   (let
3       <<kahua-subgraphs>>
4       <<plot-kahua-graph>>
5     ))
```

Listing S18. Conditionally plot the Kahua 1 and Pāhinahina graph picture.

The source code block to identify unilaterally connected subgraphs at Kahua 1 and Pāhinahina (Listing S19) calls the `connected-component` function from the `graph.lisp` library, which returns a list of all the nodes reachable from a given node. The nodes are the two early trails, T002 and T006.

```
((dsa-levels (levels graph))
 (dsa-comps (and components
                  (list
                   (connected-component
                    graph
                    (read-from-string "T002")
                    :type :unilateral)
                   (connected-component
                    graph
                    (read-from-string "T006")
                    :type :unilateral)))))
```

Listing S19. Identify unilaterally connected subgraphs at Kahua 1 and Pāhinahina.

The source code block to plot the Kahua 1 and Pāhinahina detailed study area graph picture (Listing S20) is similar to the source code block used to plot Figure 6 (Listing S12), except that, for aesthetic reasons, it sets an edge attribute to render arcs in gray, instead of black.

```
(to-dot-file graph out-file
             :attributes (list (cons :bgcolor "white")
                               (cons :aspect 2.5))
             :edge-attrs (list (cons :color (constantly "grey")))
             :node-attrs (list
                          (cons :colorscheme
                                (constantly brewer-color))
                          (cons :fillcolor
                                (lambda (n)
                                  (+ 1 (gethash n dsa-levels))))
                          (cons :fontcolor (lambda (n)
                                             (if (<= label-break
                                                     (gethash n dsa-levels))
                                                 1 "\black")))
                          (cons  :shape (lambda (n)
                                          (if (string= "T" (string n)
                                                       :start2 0
                                                       :end2 1)
                                             "box" "oval")))
                          (cons :style (constantly "filled")))
             :subgraphs (and components
                             (mapcar (lambda (x)
                                       (graph-dot:make-subgraph
                                        :attributes '(("color" . "red")
                                                      ("label" . "" ))
                                        :node-list x)) dsa-comps)))
```

Listing S20. Plot the Kahua 1 and Pāhinahina detailed study area graph picture.

References

1. Gentleman R, Temple Lang D (2007) Statistical Analyses and Reproducible Research. Journal of Computational and Graphical Statistics 16: 1–23.

2. Delescluse M, Franconville R, Joucla S, Lieury T, Pouzat C (2012) Making neurophysiological data analysis reproducible. why and how? Journal of Physiology (Paris) 106: 159–170.

3. Schulte E, Davison D (2011) Active documents with Org-mode. Computing in Science and Engineering 13: 2–9.

4. Schulte E, Davison D, Dye T, Dominik C (2012) A multi-language computing environment for literate programming and reproducible research. Journal of Statistical Software 46: 1–24.

5. Miller SD, Pushkarev VV, Gellman AJ, Kitchin JR (2013) Simulating temperature programmed desorption of oxygen on pt(111) using DFT derived coverage dependent desorption barriers. Topics in Catalysis : 1–12.

6. Knuth DE (1984) Literate programming. The Computer Journal 27: 97–111.

7. Ladefoged TN, Graves MW (2008) Variable development of dryland agriculture in Hawai‘i: A fine-grained chronology from the Kohala Field System, Hawai‘i Island. Current Anthropology 49: 771–802.
