# Supplementary material for: Structure and Growth of the Leeward Kohala Field System: An Analysis with Directed Graphs
Source: PLoS One. 2014 Jul 24;9(7):e102431. doi: 10.1371/journal.pone.0102431 (PMC4109926; doi:10.1371/journal.pone.0102431)

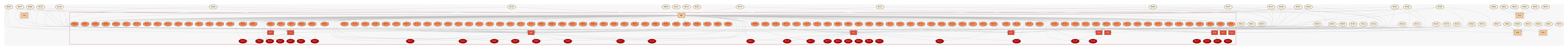

Supplement: Figure S2 — Directed graph of the agricultural walls and trails in the Lapakahi detailed study area. (PDF) [file pone.0102431.s002.pdf]
